# VB_12_Path for Accurate Metagenomic Profiling of Microbially Driven Cobalamin Synthesis Pathways

**DOI:** 10.1128/mSystems.00497-21

**Published:** 2021-06-01

**Authors:** Jiayin Zhou, Xiaoli Yu, Jihua Liu, Wei Qin, Zhili He, David Stahl, Nianzhi Jiao, Jizhong Zhou, Qichao Tu

**Affiliations:** a Institute of Marine Science and Technology, Shandong University, Qingdao, China; b Joint Laboratory for Ocean Research and Education of Dalhousie University, Shandong University and Xiamen University, Qingdao, China; c Environmental Microbiomics Research Center, School of Environmental Science and Engineering, Southern Marine Science and Engineering Guangdong Laboratory (Zhuhai), Sun Yat-sen University, Guangzhou, China; d Department of Microbiology and Plant Biology, University of Oklahoma, Norman, Oklahoma, USA; e Department of Civil and Environmental Engineering, University of Washington, Seattle, Washington, USA; f Institute of Marine Microbes and Ecospheres, Xiamen University, Xiamen, China; g Earth and Environmental Sciences, Lawrence Berkeley National Laboratory, Berkeley, California, USA; Institute of Urban Environment, Chinese Academy of Sciences

**Keywords:** cobalamin synthesis, database, functional gene, shotgun metagenome

## Abstract

Cobalamin (vitamin B12; VB_12_) is an indispensable nutrient for all living entities in the Earth’s biosphere and plays a vital role in both natural and host environments. Currently in the metagenomic era, gene families of interest are extracted and analyzed based on functional profiles by searching shotgun metagenomes against public databases. However, critical issues exist in applying public databases for specific processes such as VB_12_ biosynthesis pathways. We developed a curated functional gene database termed VB_12_Path for accurate metagenomic profiling of VB_12_ biosynthesis gene families of microbial communities in complex environments. VB_12_Path contains a total of 60 VB_12_ synthesis gene families, 287,731 sequences, and 21,154 homology groups, and it aims to provide accurate functional and taxonomic profiles of VB_12_ synthesis pathways for shotgun metagenomes and minimize false-positive assignments. VB_12_Path was applied to characterize cobalamin biosynthesis gene families in human intestines and marine environments. The results demonstrated that ocean and human intestine had dramatically different VB_12_ synthesis processes and that gene families belonging to salvage and remodeling pathway dominated human intestine but were lowest in the ocean ecosystem. VB_12_Path is expected to be a useful tool to study cobalamin biosynthesis processes via shotgun metagenome sequencing in both environmental and human microbiome research.

**IMPORTANCE** Vitamin B12 (VB_12_) is an indispensable nutrient for all living entities in the world but can only be synthesized by a small subset of prokaryotes. Therefore, this small subset of prokaryotes controls ecosystem stability and host health to some extent. However, critical accuracy and comprehensiveness issues exist in applying public databases to profile VB_12_ synthetic gene families and taxonomic groups in complex metagenomes. In this study, we developed a curated functional gene database termed VB_12_Path for accurate metagenomic profiling of VB_12_ communities in complex environments. VB_12_Path is expected to serve as a valuable tool to uncover the hidden microbial communities producing this precious nutrient on Earth.

## INTRODUCTION

Cobalamin (vitamin B12; VB_12_) is an indispensable nutrient for all living entities in the Earth’s biosphere (1, 2) and plays crucial roles in natural ecosystems and host environments such as human gut (3, 4) and nematode (5). Since *in vivo* biosynthesis of cobalamin is an energetically expensive process involving the expression of more than 30 enzymatic steps via aerobic or anaerobic pathways, cobalamin can only be synthesized by a small subset of prokaryotic groups (6), while other microorganisms need exogenous supplies. Exogeneous cobalamin can be obtained mainly through two approaches: direct interactions with producers and decomposition of cells containing cobalamin (7). The production, transformation, and circulation of cobalamin play a fundamental role in structuring microbial community diversity and activity as well as strengthening interactions between microorganisms. For example, while phytoplankton accounts for about half of the global primary production, most of the eukaryotic phytoplankton in the surface ocean are VB_12_ auxotrophs (8), and several studies have confirmed that the availability of cobalamin can limit the growth and activity of phytoplankton and thus primary productivity (7). Cobalamin also has potential major effects on biogeochemical cycling driven by microorganisms and a wide variety of general microbial processes involved in gene expression regulation (9), CO_2_ fixation, DNA replication and repair, and synthesis of amino acids (10). Recent research on *Nitrospira* spp. has shown that there are close links between cobalamin production and aerobic nitrogen cycle (11). Also, in human beings, cobalamin functions as the “anti-pernicious anemia factor” and holds a certain significance of resisting the development of pernicious anemia in animals, shaping the composition of human gut microbial communities (4). Over 83% of sequenced human gut microbial strains possess enzymes that are dependent on VB_12_ (3). The human gut microbiome is also expected to synthesize different forms of cobalamin as the essential micronutrients (1). In addition, vitamin B_12_ deficiency could cause humans to produce methylmalonic aciduria and homocysteinemia that eventually lead to devastating diseases and affect the human nervous system (5). Therefore, revealing the taxonomic and functional composition of microbial communities involved in cobalamin biosynthesis and transformation in various ecosystems is of crucial importance to understand the cobalamin-based microbial interdependencies that sustain the ecosystem’s multifunctioning, stability, and human/host health.

Over the past years, efforts and progress have been made to expand our understanding and knowledge of the microbial processes that are responsible for cobalamin biosynthesis and transformation. For example, advances in methodology allow direct measurements of cobalamin in various environmental samples (12). The employment of analytical chemistry techniques helps identify genes involved in cobalamin circulating processes (13). It is known that cobalamin is synthesized exclusively by a subset of bacteria and archaea (10) that requires more than 30 enzymatic steps involving a total of 60 gene families, including the *BtuBFCD* transporter (3) and ATP-binding cassette (ABC) transport system. The synthesis of cobalamin can be divided into two categories, namely, *de novo* pathway and salvage and remodeling pathway. The *de novo* pathway can be further divided into aerobic pathway and anerobic pathway according to the timing of cobalt insertion and the oxygen requirements (11). Most reactions of aerobic and anaerobic pathways share similar metabolic steps, making it difficult to distinguish corresponding gene families due to their high homology. For instance, most methyltransferases involved in peripheral methylation reactions have been confirmed with high sequence similarity (14). Genes belonging to *cysG* and *cobA* are also highly homologous due to similar functional domains (15). Traditionally, corresponding gene families belonging to aerobic and anaerobic pathways were respectively named using different prefixes as *cob* and *cbi* from different organisms (16). Notably, the corresponding *cbi* and *cob* gene families involved in aerobic and anaerobic pathways, respectively, are usually mixed together as the same orthologous group in public orthology databases.

Recent advances in high-throughput metagenome sequencing have made community-level functional and taxonomic profiling of complex microbial communities a routine application in microbial ecology studies (17). Microbial species encoding proteins of VB_12_ biosynthesis pathways and their relationships with other organisms in complex natural ecosystems and host environments can also be studied in an elegant manner, such as via the combination of metagenomic and chemical characterization of cobalamin production in soils (11). To do so, metagenome sequences are searched against public ortholog databases, e.g., KEGG (Kyoto Encyclopedia of Genes and Genome) (18), eggNOG (evolutionary genealogy of genes: Nonsupervised Orthologous Groups) (19), and COG (Clusters of Orthologous Groups of proteins) (20), based on which gene families of interest could be extracted and subjected to further detailed analyses. However, as we reported previously (21), there are many challenges for accurate profiling of functional gene families with public ortholog databases. First, orthologous groups in most orthology databases are in fact sequence clusters based solely on sequence identities and are therefore usually mixtures of multiple different gene families. Second, our most recent knowledge and understanding of these specific gene families and pathways are usually not reflected in these public ortholog databases. Third, searching against large-scale comprehensive databases is computationally expensive and time-consuming, while searching against customized databases containing extracted gene families can easily lead to high false-positive assignments due to small database issues. Taking VB_12_ synthesis gene families for example, gene families belonging to aerobic and anaerobic processes are usually mixed together in the same orthologous group and thus can hardly be separated in database searching. These issues generally lead to inaccurate and less-useful profiling of functional gene families at the cost of extensive computational resources and time.

In this study, we aimed to develop a curated functional gene database as well as tool sets (VB_12_Path) for metagenomic profiling of gene families involved in VB_12_ biosynthesis pathways. VB_12_Path held multiple advantages compared to public ortholog databases, such as accurate definition of gene families and sequences, minimum false-positive assignment by comprehensively including homologous gene families, clear separation of homologous gene families in aerobic and anaerobic processes, and both functional and taxonomic profiling tool sets for VB_12_ gene families. VB_12_Path was applied to profile both functional gene families and taxonomic compositions of VB_12_ biosynthesis pathways in human gut and ocean environments. The results demonstrated that VB_12_Path is a useful tool for analyzing VB_12_ biosynthesis pathways of microbial communities from different environments via metagenome sequencing.

## RESULTS

### Summary of gene families involved in VB_12_ synthesis pathways.

For VB_12_Path, a total of 60 key gene families involved in VB_12_ biosynthesis were selected, targeting metabolic processes of VB_12_ biosynthesis pathways in different microorganisms, including *de novo* biosynthesis pathways (aerobic and anaerobic) and salvage and remodeling pathways (Table S1). Since multiple gene families were shared by these different pathways, we further divided the complex VB_12_ pathways into five different processes, including precorrin-2 synthesis, aerobic, anaerobic, salvage and remodeling, and post-AdoCbi-P processes (Fig. S1). A total of 4,079, 168,630, and 287,731 sequences were collected for the seed database, the core database, and the full database, respectively. In addition to targeted gene families, 21,154 homologous groups were also identified and included in VB_12_Path, with the purpose to minimize false-positive assignments. Among these homologous groups, 650 were from arCOG database, 770 from COG database, 17,546 from eggNOG database, and 2,188 from KEGG database (Table 1). To illustrate the extent that “small database” may affect metagenomic profiling, five randomly selected TARA Oceans metagenomes were profiled against the core and full database of VB_12_Path. The results suggested that about 52% of sequences targeted by VB_12_ gene families in the core database could be better assigned to their homologs in the full database (Fig. S2), suggesting that small databases not considering their homologs could be a critical issue for functional assignment in metagenomics.

**TABLE 1:** Summary of representative sequences and nontarget homologous groups for selected gene families in VB_12_Path

Pathway	Gene (sub)family	Annotation	No. of core database sequences	No. of full database sequences	No. of orthology groups with homologous sequences			
					arCOG	COG	eggNOG	KEGG
Precorrin-2 synthesis	*cobA*^*a*^	Uroporphyrinogen-III c-methyltransferase	3,183	10,596	22	24	604	65
	*hemA*	Glutamyl-tRNA reductase	18,014	21,723	64	75	1,590	172
	*hemB*	Delta-aminolevulinic acid dehydratase	5,243	13,701	8	17	256	33
	*hemC*	Porphobilinogen deaminase	21,924	28,373	57	61	1,487	202
	*hemD*	Porphyrin biosynthesis protein HemD	5	26	NA^*b*^	NA	2	0
	*hemL*	Glutamate-1-semialdehyde 2,1-aminomutase	23,309	29,979	39	51	973	162
	*cysG*	Siroheme synthase	577	3,654	3	4	91	25
	*gltX*	Glutamate-tRNA ligase	35,259	46,056	88	92	2,134	251
	Sum		107,514	154,108	281	324	7,137	910	
Aerobic	*cobB*	Hydrogenobyrinate a,c-diamide synthase	2,231	5,182	10	17	571	64
	*cobC*-*beta*	Threonine-phosphate decarboxylase	85	1,260	1	0	20	4
	*cobD*^*a*^	Threonine-phosphate decarboxylase	142	696	2	3	15	6
	*cobF*	Precorrin-6A synthase	282	1,580	NA	1	8	1
	*cobG*	Precorrin-3B synthase	80	443	NA	0	14	4
	*cobH*	Precorrin-8X methylmutase	958	3,192	2	7	73	20
	*cobI*	Precorrin-2 C(20)-methyltransferase	628	2,910	3	7	75	14
	*cobJ*	Precorrin-3B C(17)-methyltransferase	1,138	4,027	6	9	209	15
	*cobK*	Precorrin-6A reductase	405	2,444	1	5	88	16
	*cobL*	Precorrin-6Y C(5,15)-methyltransferase	590	3,139	4	9	131	27
	*cobM*	Precorrin-4 C(11)-methyltransferase	1,975	4,619	2	6	101	18
	*cobN*	Aerobic cobaltochelatase subunit CobN	328	2,987	2	8	234	26
	*cobO*^*a*^	Corrinoid adenosyltransferase	1,167	3,764	0	4	36	4
	*cobP*^*a*^	Bifunctional adenosylcobalamin biosynthesis protein CobP	364	3,133	1	4	41	9
	*cobQ*	Cobyric acid synthase	4,815	9,823	25	53	692	97
	*cobR*	Cob(II)yrinic acid a,c-diamide reductase	1	8	NA	NA	1	NA
	*cobS*-*co*	Aerobic cobaltochelatase subunit CobS	269	1,394	NA	0	14	2
	*cobT*-*co*	Aerobic cobaltochelatase subunit CobT	210	1,437	3	2	59	4
	Sum		15,668	52,038	62	135	2,382	331	
Anaerobic	*cobA*^*a*^	Uroporphyrinogen-III c-methyltransferase	3,183	10,596	22	24	604	65
	*cobD*^*a*^	Threonine-phosphate decarboxylase	142	696	2	3	15	6
	*cobU*-*ade*^*a*^	Bifunctional adenosylcobalamin biosynthesis protein CobU	441	1,305	1	0	15	0
	*cbiA*	Cobyrinate a,c-diamide synthase	3,282	5,293	3	17	349	50
	*cbiB*^*a*^	Cobalamin biosynthesis protein CbiB	273	900	0	0	2	0
	*cbiC*	Cobalt-precorrin-8 methylmutase	80	986	0	0	7	3
	*cbiD*	Cobalt-precorrin-5B C(1)-methyltransferase	7,485	9,637	32	43	673	113
	*cbiE*	Cobalt-precorrin-7 C(5)-methyltransferase	30	244	0	1	2	1
	*cbiF*	Cobalt-precorrin-4 C(11)-methyltransferase	472	655	1	5	42	11
	*cbiG*	Cobalt-precorrin-5A hydrolase	401	827	1	0	8	2
	*cbiH*	Cobalt-factor III C(17)-methyltransferase	10	423	1	5	9	4
	*cbiJ*	Cobalt-precorrin-6A reductase	33	100	0	0	2	0
	*cbiK*	Sirohydrochlorin cobaltochelatase	196	230	1	1	7	1
	*cbiL*	Cobalt-precorrin-2 C(20)-methyltransferase	164	252	0	0	5	0
	*cbiP*	Cobyric acid synthase	1	1	NA	0	0	0
	*cbiT*	Cobalt-precorrin-6B C(15)-methyltransferase	377	939	1	0	23	7
	*cbiX*	Sirohydrochlorin cobaltochelatase	339	1,285	4	5	43	8
	*btuR*	Cob(I)alamin adenosyltransferase	14	32	0	0	3	1
	*pduX*	l-threonine kinase	61	131	NA	0	2	0
	Sum		16,984	34,532	69	104	1,811	272	
Salvage and remodeling	*btuB*	Vitamin B_12_ transporter BtuB	1,857	2,328	NA	8	66	10
	*btuC*	Vitamin B_12_ import system permease protein BtuC	1,610	2,292	8	11	297	41
	*btuD*	Vitamin B_12_ import ATP-binding protein BtuD	1,486	2,232	22	49	139	115
	*btuF*	Vitamin B_12_-binding protein	1,616	2,427	1	2	53	12
	*cobA*^*a*^	Uroporphyrinogen-III c-methyltransferase	3,183	10,596	22	24	604	65
	*cobO*^*a*^	Corrinoid adenosyltransferase	1,167	3,764	0	4	36	4
	*cobP*^*a*^	Bifunctional adenosylcobalamin biosynthesis protein CobP	364	3,133	1	4	41	9
	*cobU*-*ade*^*a*^	Bifunctional adenosylcobalamin biosynthesis protein CobU	441	1,305	1	0	15	0
	*cobY*	Adenosylcobinamide phosphate guanylyltransferase	9	81	0	0	0	0
	*cbiB*^*a*^	Cobalamin biosynthesis protein CbiB	273	900	0	0	2	0
	*cbiZ*	Adenosylcobinamide amidohydrolase	13	105	1	1	2	1
	*eutT*	Ethanolamine utilization cobalamin adenosyltransferase	64	127	NA	0	6	0
	*pduO*^*a*^	Corrinoid adenosyltransferase	134	885	0	2	10	2
	Sum		12,217	30,175	56	105	1,271	259	
Post-AdoCbi-P	*cobC*-*ado*	Adenosylcobalamin phosphatase	53	142	1	2	4	8
	*cobP*^*a*^	Bifunctional adenosylcobalamin biosynthesis protein CobP	364	3,133	1	4	41	9
	*cobS*-*gdp*	Adenosylcobinamide GDP ribazoletransferase	12,009	25,088	176	77	3,920	322
	*cobT*-*alpha*	Nicotinate-nucleotide-dimethylbenzimidazole phosphoribosyltransferase	11,555	20,236	52	76	2,252	215
	*cobU*-*alpha*	Nicotinate-nucleotide-dimethylbenzimidazole phosphoribosyltransferase	1	4	NA	NA	0	0
	*cobU*-*ade*^*a*^	Bifunctional adenosylcobalamin biosynthesis protein CobU	441	1,305	1	0	15	0
	*cobV*	Adenosylcobinamide GDP ribazoletransferase	1	2	NA	NA	0	0
	*pduS*	Cob(II)alamin reductase	1	217	NA	1	2	1
	*pduO*^*a*^	Corrinoid adenosyltransferase	134	885	0	2	10	2
	*fre*	NAD(P)H-flavin reductase	56	77	NA	1	5	3
	*ubiB*	Aquacobalamin reductase	1	1	NA	NA	NA	NA
	*bluB*	5,6-dimethylbenzimidazole synthase	1,323	2,101	1	4	78	16
	Sum		25,939	53,191	232	167	6,328	576	
	Total		178,322	324,044	700	835	18,929	2,348	

a Gene families appearing in multiple pathways.

b NA, not available.

10.1128/mSystems.00497-21.1 TABLE S1 Summary of VB_12_ synthesis genes with corresponding protein name. Download Table S1, DOCX file, 0.02 MB. Copyright © 2021 Zhou et al. 2021 Zhou et al. https://creativecommons.org/licenses/by/4.0/ This content is distributed under the terms of the Creative Commons Attribution 4.0 International license.

10.1128/mSystems.00497-21.3 FIG S1 Cobalamin biosynthesis pathway and related gene families. The whole VB_12_ biosynthesis pathway was divided into five different processes, including precorrine-2 synthesis, aerobic pathway, anaerobic pathway, salvage and remodeling pathway, and post-AdoCbi-P pathway. For each step, the metabolites and their corresponding protein coding gene families were illustrated. Download FIG S1, TIF file, 2.9 MB. Copyright © 2021 Zhou et al. 2021 Zhou et al. https://creativecommons.org/licenses/by/4.0/ This content is distributed under the terms of the Creative Commons Attribution 4.0 International license.

10.1128/mSystems.00497-21.4 FIG S2 Illustration of the “small database issue” using the core and full databases of VB_12_Path. The core VB_12_Path only contains VB_12_ biosynthesis gene families, while the full database contains both VB_12_ biosynthesis gene families and their homologs. Five TARA Oceans metagenomic datasets were used for the evaluation here. The results showed that about 52% sequences were better mapped to VB_12_ gene homolog if homologous sequences were included in the database, suggesting critical “small database issue” in metagenomic profiling. Download FIG S2, TIF file, 1.2 MB. Copyright © 2021 Zhou et al. 2021 Zhou et al. https://creativecommons.org/licenses/by/4.0/ This content is distributed under the terms of the Creative Commons Attribution 4.0 International license.

### Precorrin-2 synthesis processes.

Eight gene families are known to be responsible for δ-aminolevulinate (ALA) formation and precorrin-2 synthesis from ALA. These gene families are shared by both aerobic and anaerobic biosynthesis pathways. Three gene families, including *hemA*, *hemL*, and *gltX*, are responsible for ALA synthesis via C4 and C5 pathways. Conversion of ALA to uroporphyrinogen-III is carried out by enzymes encoded by *hemC*, *hemB*, and *hemD*. Precorrin-2 is then synthesized via methylation of uroporphyrinogen-III at positions C2 and C7. This process is catalyzed by enzymes encoded by *cobA* and *cysG*. A total of 154,108 sequences and 8,652 homologous groups were present for these eight gene families in VB_12_Path.

### Aerobic pathway.

Gene families encoding enzymes that convert precorrin-2 to adenosylcobinamide phosphate were selected for aerobic biosynthesis pathway. A total of 18 gene families were recruited, including *cobI*, *cobG*, *cobJ*, *cobM*, *cobF*, *cobK*, *cobL*, *cobH*, *cobB*, *cobN*, *cobS-co*, *cobT-co*, *cobR*, *cobO*, *cobQ*, *cobC-beta*, *cobD*, and *cobP*. Among them, conversion of hydrogenobyrinic acid a,c-diamide to cob(II)yrinic acid a,c-diamide through cobalt chelation is carried out by enzymes encoded by *cobN*, *cobS*, and *cobT*. The aerobic and anaerobic pathways converge after coby(II)rinic acid a,c-diamide. Adenosylcobyric acid is then formed via adenylation and amidation of cob(I)yrinic acid a,c-diamide. This process is catalyzed by enzymes encoded by *cobR*, *cobO*, and *cobQ*, and (*R*)-1-amino-2-propanol is directly attached at the f position of the carboxyl group of adenosylcobyric acid and converted to adenosylcobinamide phosphate (AdoCbi-P). This process is catalyzed by proteins α and β encoded by *cobC-beta* and *cobD*. A total of 52,038 sequences and 2,910 homologous groups were present for these 18 gene families in VB_12_Path.

### Anaerobic pathway.

Gene families encoding enzymes that convert sirohydrochlorin to adenosylcobinamide phosphate were selected for anaerobic biosynthesis pathway. A total of 19 gene families were recruited, including *cbiK*, *cbiX*, *cbiL*, *cbiH*, *cbiF*, *cbiG*, *cbiD*, *cbiJ*, *cbiE*, *cbiT*, *cbiC*, *cbiA*, *btuR*, *cobA*, *cbiP*, *cobU-ade*, *pduX*, *cobD*, and *cbiB*. Enzymes encoded by *cobA* and *cbiP* are responsible for yielding adenosylcobyric acid from cob(II)yrinic acid a,c-diamide via adenylation and amidation reactions. Conversion of l-threonine *O*-phosphate to (*R*)-1-amino-2-propanol *O*-2-phosphate is carried out by enzymes encoded by *pduX* and *cobD*. (*R*)-1-amino-2-propanol *O*-2-phosphate is then directly attached at the f position of the carboxyl group of adenosylcobyric acid and converted to AdoCbi-P. This process is catalyzed by enzymes encoded by *cobC-beta* and *cobD.* A total of 34,532 sequences and 2,256 homologous groups were present for these 19 gene families in VB_12_Path.

### Salvage and remodeling pathway.

The salvage pathway is a cost-effective way for bacteria and archaea to obtain cobalamin without consuming much energy. Gene families encoding enzymes that synthesize adenosylcobinamide phosphate (AdoCbl-P) from cobinamide were screened for salvage and remodeling pathway. A total of 13 gene families were recruited, including *btuB*, *btuF*, *btuC*, *btuD*, *cobA*, *cobO*, *eutT*, *pduO*, *cobP*, *cobU-ade*, *cobY*, *cbiB*, and *cbiZ*. Among these, the *BtuBFCD* transporter system in bacteria has received much attention from biologists. Cobinamide is transported into the cell via *BtuBFCD* transporter systems, which are encoded by *btuB*, *btuF*, *btuC*, and *btuD*. Cobinamide is adenosylated by ATP:co(I)rrinoid adenosyltransferases and converted to adenosylcobinamide (AdoCbi). This process is catalyzed by enzymes encoded by *cobA*, *eutT*, *pduO*, and *cobO*, and then AdoCbi is converted to AdoCbi-P by enzymes encoded by *cobU-ade*, *cobP*, *cbiB*, *cbiZ*, and *cobY*. A total of 30,175 sequences and 1,691 homologous groups were present for these 13 gene families in VB_12_Path.

### Post-AdoCbi-P pathway.

Twelve gene families were responsible for adenosylcobalamin (AdoCbl) synthesis from AdoCbi-P. These gene families were shared by both *de novo* and salvage and remodeling biosynthesis pathways. Conversion of AdoCbi-P to adenosylcobalamin 5′-phosphate (AdoCbl 5′-P) is carried out by enzymes encoded by *cobU-ade*, *cobP*, and *cobS-gdp*. Another alternative step about the synthesis of AdoCbl 5′-P is through 5,6-dimethylbenzimidazole (DMB). Three gene families, including *bluB*, *fre*, and *ubiB*, are responsible for DMB synthesis. Then, DMB is converted to AdoCbl 5′-P by enzymes encoded by *cobU-alpha*, *cobT-alpha*, and *cobV*. Finally, AdoCbl is synthesized via dephosphorylation of AdoCbl 5′-P. This process is catalyzed by enzymes encoded by *cobC-ado*. A total of 53,191 sequences and 7,303 homologous groups were present for these 12 gene families in VB_12_Path.

### Comparative analysis of VB_12_ gene families in VB_12_Path and public orthology databases.

**FIG 1:**
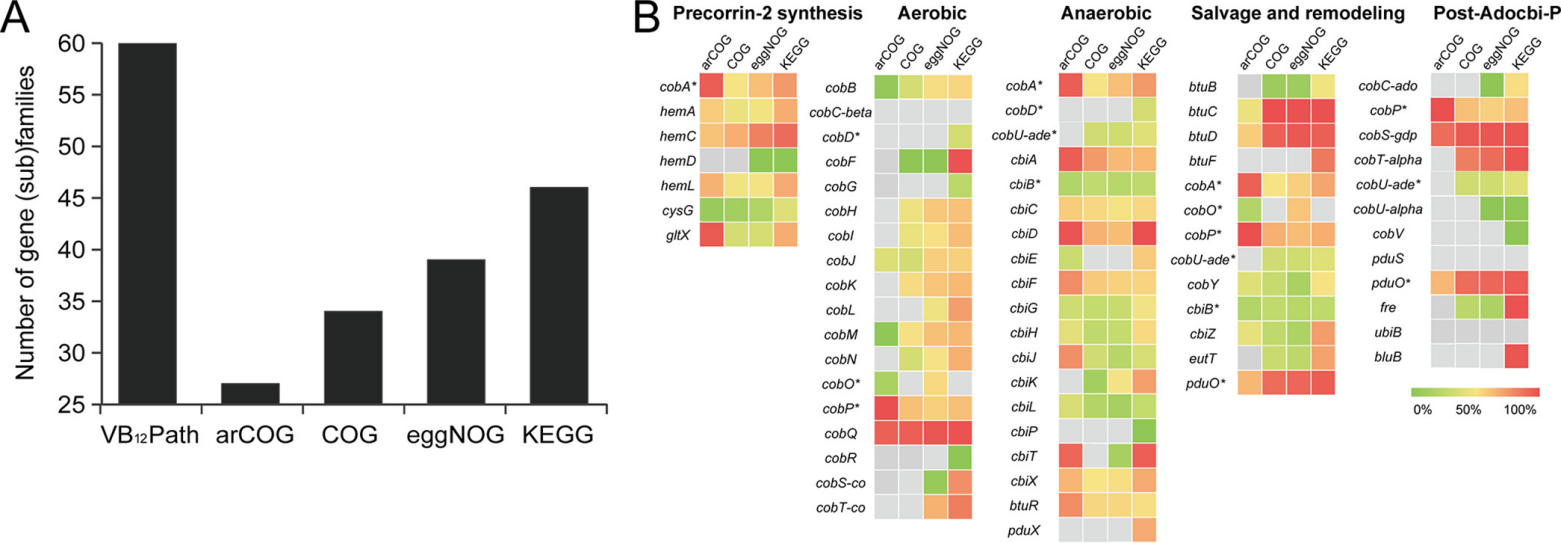
VB_12_ synthesis gene (sub)families in VB_12_Path and other orthologous databases. (A) The number of gene (sub)families or orthologous groups responsible for VB_12_ biosynthesis detected in different databases. (B) The accuracy of gene families defined in orthologous databases. Accuracy was measured by the percentage of sequences belonging to the selected VB_12_ synthesis gene (sub)families in corresponding orthology groups in public databases. Gene families appearing in multiple pathways are marked with an asterisk. Although separately plotted, some gene families may belong to a single orthologous group in public orthology databases.

VB_12_Path was compared with other major public orthology databases in terms of coverage, accuracy, and specificity. Mapping information between VB_12_ gene families and orthologous groups was obtained during the full database construction process. First, the coverage of VB_12_ gene (sub)families in VB_12_Path and other databases was compared to evaluate the integrity of VB_12_ biosynthesis pathways in different databases. The result showed that a total of 60 gene (sub)families were included in VB_12_Path, while the number of orthologous groups involved in VB_12_ biosynthesis found in arCOG, COG, eggNOG, and KEGG orthology databases was 27, 34, 39, and 46, respectively (Fig. 1A). Gene families such as *pduO*, *pduS*, and *ubiB* that were also of great importance were missing in these four orthology databases. Second, the accuracy of VB_12_ gene families in public orthology databases was analyzed (Fig. 1B). Surprisingly, only a few gene families, including *cobQ*, *hemA*, *hemB*, *hemC*, and *hemL*, were represented with high accuracy in these public databases, i.e., the corresponding orthologous groups were composed by sequences belonging to these gene families. Such inaccuracy in gene family definition and representative sequences would eventually result in inaccurate functional profiles of VB_12_ gene families when annotating shotgun metagenomes using these orthology databases. Third, homologous gene families were rarely distinguished in these public orthology databases, while they usually played different functions in VB_12_ biosynthesis pathways. One such example was *cysG* and *cobA*, which were always mixed together as one orthologous group in almost all orthology databases due to the high sequence similarity shared by these genes. Other homologous gene families with similar issues included *cobR* and *cbiH*, *cobH* and *cbiC*, *cobM* and *cbiF*, *cobJ* and *cbiH*, etc. These results suggested that VB_12_Path was a comprehensive, accurate, and discriminative functional gene database of VB_12_ biosynthesis gene families.

The accuracy of VB_12_Path was further illustrated using metagenomic data and an artificial data set. The results were compared against one of the best current alternatives, the KEGG database. In the metagenomic data set, the relative abundances of VB_12_ gene families identified by KEGG were generally higher than those identified by VB_12_Path (Fig. S3). This is because, in addition to VB_12_ biosynthesis genes, their homologs were always included in the KEGG orthologous groups, thereby leading to higher relative abundances of VB_12_ gene families. In addition, a high number of gene families were mixed in a single orthologous group, leading to ambiguous interpretation of the results. In the artificial data set, a total of 270 protein sequences were retrieved from the NCBI GenBank database, of which 143 belonged to VB_12_ biosynthesis gene families, 57 were their homologs, and the remaining 70 were unrelated sequences. As a result, all VB_12_ biosynthesis genes were correctly assigned to their targeting gene families in VB_12_Path, while their homologs and unrelated sequences were not assigned. For KEGG, however, both false-positive (23%) and false-negative (3%) observations were found for VB_12_ biosynthesis gene families (Table S2). A high rate of false positives (22%) was also observed for unrelated sequences, though they are not related to VB_12_ biosynthesis. Such accuracy issues in public orthology databases (e.g., KEGG) should be due to the mixed status of targeted gene families and their homologs in the same orthologous group. Notably, considering the extremely high complexity of metagenome sequences, false positives cannot be completely avoided by any methodologies but can only be minimized with our best current knowledge.

10.1128/mSystems.00497-21.2 TABLE S2 Summary of mapped sequences of an artificial data set. Red denotes false positives and blue denotes false negatives. Download Table S2, XLSX file, 0.04 MB. Copyright © 2021 Zhou et al. 2021 Zhou et al. https://creativecommons.org/licenses/by/4.0/ This content is distributed under the terms of the Creative Commons Attribution 4.0 International license.

10.1128/mSystems.00497-21.5 FIG S3 Comparative evaluation of metagenomic profiling of VB_12_ gene families using KEGG (A) and VB_12_Path (B). A total of 36 gene families were illustrated. The relative abundances of VB_12_ gene families identified by KEGG were generally higher than those detected by VB_12_Path. This is because in addition to VB_12_ biosynthesis genes, their homologs are always included in the KEGG orthologous groups, thereby leading to higher relative abundances of VB_12_ gene families. Download FIG S3, TIF file, 2.1 MB. Copyright © 2021 Zhou et al. 2021 Zhou et al. https://creativecommons.org/licenses/by/4.0/ This content is distributed under the terms of the Creative Commons Attribution 4.0 International license.

### VB_12_ biosynthesis pathways in sequenced microbial genomes.

**FIG 2:**
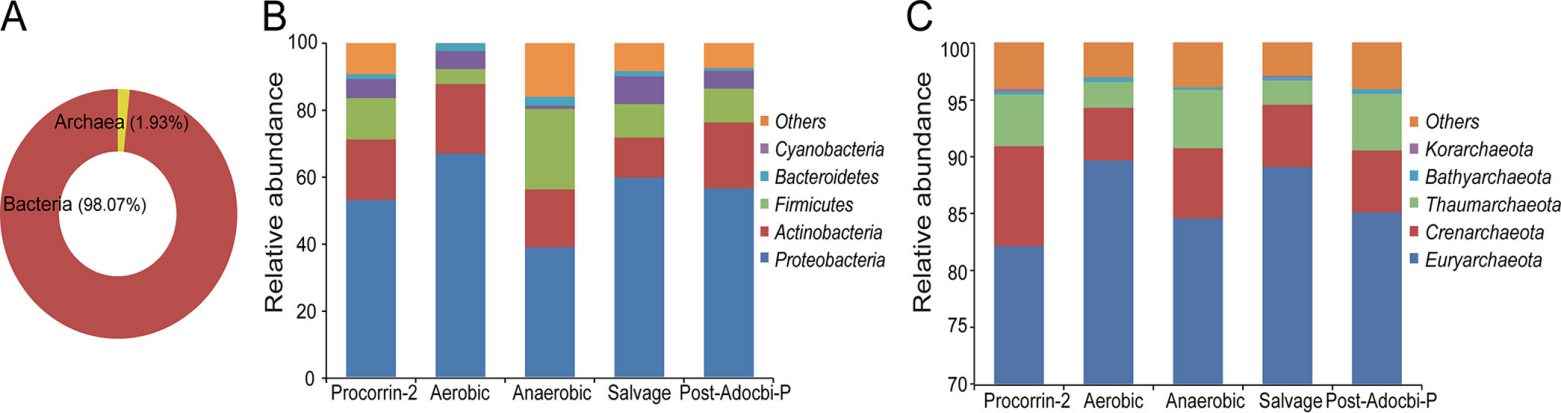
Situation of VB_12_ synthesis pathways in sequenced microbial genomes. (A) The current sequenced microbial genomes indicate that the cobalamin synthesis pathway is mainly contributed by bacterial genes. The taxonomic composition and relative abundance distribution of bacterial (B) and archaeal (C) gene families in different VB_12_ synthesis processes at the phylum level are also displayed.

Sequenced microbial genomes from the NCBI RefSeq can be mapped to the VB_12_Path to investigate how VB_12_ biosynthesis gene families are distributed across different microbial lineages. Overall, cobalamin biosynthesis pathways were distributed mainly in bacterial genomes, which accounted for approximately 98.07% of the total number of sequences (Fig. 2A). A total of 58 phyla, 95 classes, 224 orders, 494 families, and 2,488 genera were detected to have VB_12_ biosynthesis-related gene families in their genomes. Among these, in the bacterial domain, *Proteobacteria* (57.24%), *Actinobacteria* (18.16%), and *Firmicutes* (11.01%) were the most abundant phyla with VB_12_ biosynthesis gene families (Fig. 2B). At the family level, *Enterobacteriaceae* (8.04%) was the most abundant family, followed by *Pseudomonadaceae* (7.64%) and *Streptomycetaceae* (6.90%). At the genus level, *Pseudomonas* (7.57%), *Streptomyces* (6.69%), and *Vibrio* (3.37%) were the most abundant genera that had VB_12_ biosynthesis genes. A relatively small number of archaea species were found with VB_12_ biosynthesis gene families (Fig. 2A and C). Among these, *Euryarchaeota* (85.33%) had the highest abundance, followed by *Crenarchaeota* (6.20%) and *Thaumarchaeota* (4.31%). At the pathway level, precorrin-2 synthesis process was the most abundant process, with 207,401 sequences, followed by aerobic pathway (138,130 sequences), post-AdoCbi-P pathway (95,934 sequences), salvage and remodeling pathway (91,337 sequences), and anaerobic pathway (81,878 sequences). Notably, the taxonomic composition of these five pathways was generally similar at the phylum level with slight differences for aerobic pathways.

### Application of VB_12_Path to human gut and ocean metagenome analysis.

**FIG 3:**
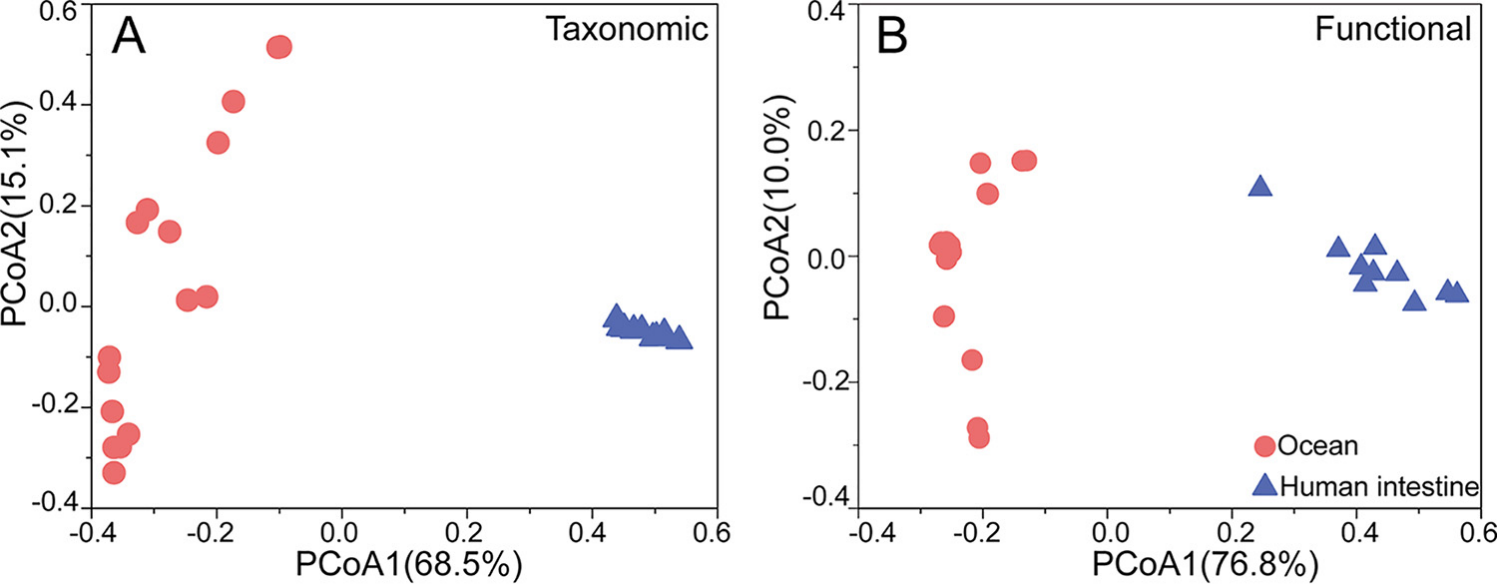
Application of VB_12_Path to characterize cobalamin synthesis gene (sub)family in marine and human intestinal environments. Principal coordinate analysis (PCoA) clustering of ocean and gut examples based on Bray-Curtis distances of (A) taxonomic composition and (B) functional genes composition. A clear separation of human intestine and ocean samples could be observed for both taxonomic and functional gene compositions.

**FIG 4:**
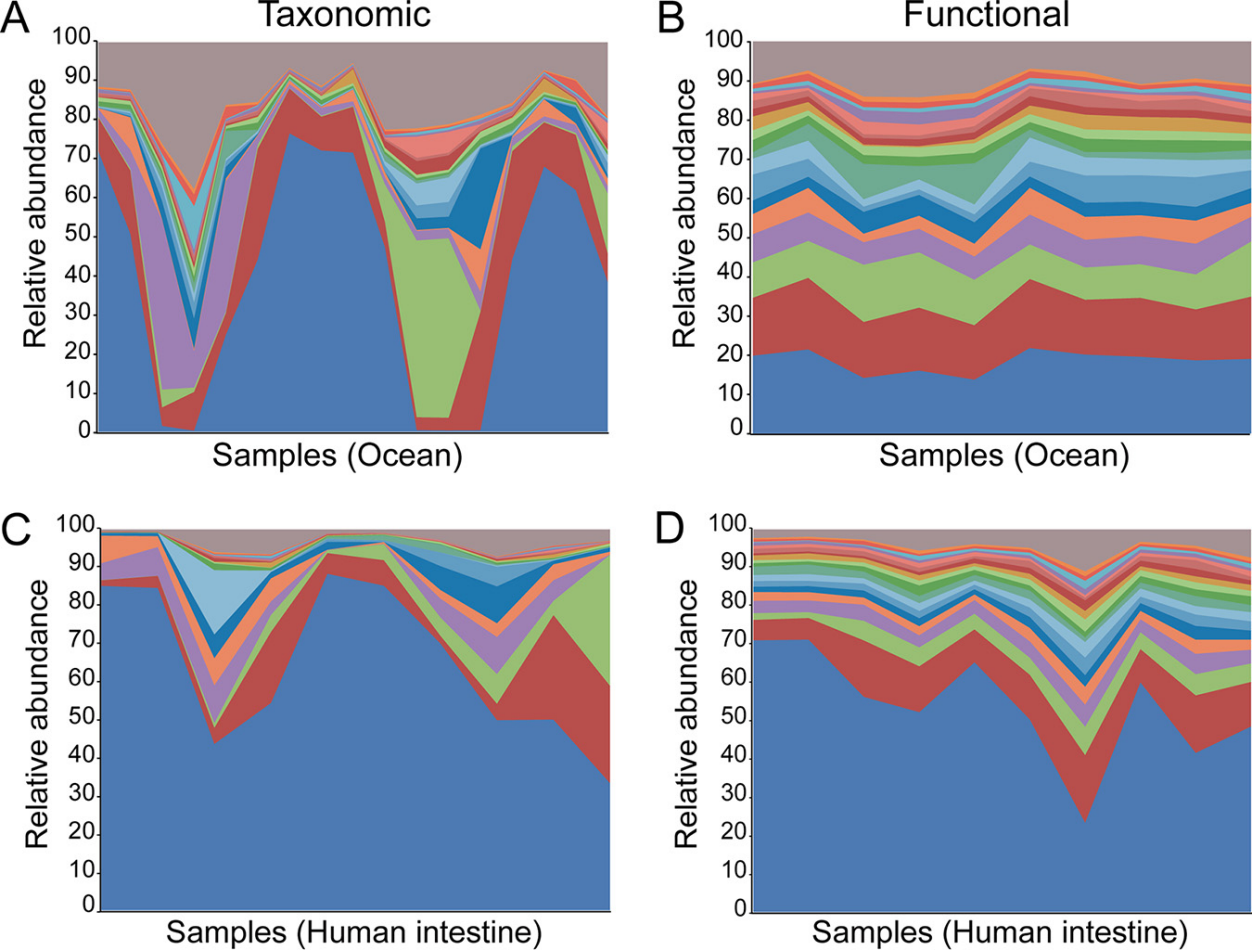
Composition and relative abundance of microbial groups at family level and gene family level in ocean (A and B) and human intestine samples (C and D). The left panels represent taxonomic compositions at family level, while the right panels represent functional gene families. Different colors refer to different species groups and gene families. Color legends are not shown in the figure because the main purpose of this figure is to describe the high variation of taxonomic compositions but stable functional gene compositions of VB_12_ synthesis pathways among different samples.

To show how VB_12_Path can be used to characterize VB_12_ biosynthesis pathways in complex metagenomic data, shotgun metagenome data sets from human gut and ocean samples were searched against VB_12_Path to profile the taxonomic composition and functional potential involved in the synthesis of cobalamin. As expected, PCoA clustering of taxonomic and functional gene profiles suggested that the VB_12_ biosynthesis microbial assemblages in human intestine and ocean had dramatically different taxonomic and functional gene compositions (Fig. 3). A total of 45 phyla, 84 classes, 187 orders, 416 families, 1,524 genera, and 5,826 species involved in VB_12_ biosynthesis were detected in marine samples. In human intestinal samples, a total of 28 phyla, 70 classes, 154 orders, 336 families, 985 genera, and 2,350 species were detected. The taxonomic composition of ocean and human intestine microbial communities associated with VB_12_ biosynthesis was dramatically different even at the phylum level. *Cyanobacteria* (40.01%), *Proteobacteria* (38.09% relative abundance), and *Bacteroidetes* (8.59%) were predominant groups in marine samples, while *Bacteroidetes* (81.19%), *Firmicutes* (12.10%), and *Proteobacteria* (0.95%) were predominant in intestinal samples. At the family level, the predominant families recovered from ocean were *Prochloraceae* (39.18%), *Pelagibacteraceae* (12.23%), and *Alteromonadaceae* (7.56%) (Fig. 4A), while in the human intestine, *Bacteroidaceae* (64.53%) was the most abundant group, followed by *Lachnospiraceae* (9.91%) and *Ruminococcaceae* (6.15%) (Fig. 4C).

Functional gene profiles for VB_12_ biosynthesis pathways were also dramatically different between ocean and human intestine samples. In ocean, gene families belonging to precorrin-2 synthesis pathway and aerobic pathway accounted for about 59% and 22% of the total sequences, respectively. Gene families belonging to salvage and remodeling pathway had the fewest sequences in ocean samples, which accounted for about 5% of the total sequences identified by VB_12_Path. In the human intestine, however, gene families involved in salvage and remodeling pathway had the most sequences, which accounted for about 60% of the identified sequences. Gene families belonging to aerobic pathway were found with the fewest assigned sequences in human intestine samples, which accounted for about 7% of the sequences. Among all VB_12_ biosynthesis gene families, *hemA*, *hemB*, *hemC*, *hemL*, and *gltX* were abundant in both marine and intestinal samples (Fig. 4B and D), while gene families including *hemD*, *ubiB*, *cobV*, *cbiP*, and *cbiZ* were rarely detected in both marine and intestine samples. Importantly, it was also noticed that microbial taxonomic groups carrying VB_12_ gene families varied highly in both human gut and ocean ecosystems, but the functional gene families were relatively stable, as observed in different angles such as ordination analysis, relative abundance, and Bray-Curtis dissimilarity indices (Fig. 3, Fig. 4, and Fig. S4). The results suggest that VB_12_Path can be used as a useful tool to explore both functional gene and taxonomic profiling of VB_12_ biosynthesis microbial communities from different environments.

10.1128/mSystems.00497-21.6 FIG S4 Community dissimilarity of microbial species and functional gene families involved in VB_12_ biosynthesis in ocean (A) and human intestine (B). The Bray-Curtis dissimilarity considering the relative abundances of microbial species and gene families was used here. Download FIG S4, TIF file, 1.6 MB. Copyright © 2021 Zhou et al. 2021 Zhou et al. https://creativecommons.org/licenses/by/4.0/ This content is distributed under the terms of the Creative Commons Attribution 4.0 International license.

## DISCUSSION

Cobalamin (VB_12_), hailed as “nature’s most beautiful cofactor” (22), is of essential importance to natural ecosystems and human health. As a micronutrient that can be synthesized by only a selective group of microbes, VB_12_ plays important roles in structuring the composition of microbial community in various ecosystems (4, 11, 23, 24), modulating various biogeochemical cycles (9, 25), and controlling the life and death of human intestinal microbes (1). A series of processes, such as ecosystem stability, microbial growth and development (5), human cardiovascular health, cognitive function (26), and intestinal microbial ecology (4, 25), are affected by the availability of VB_12_. In recent years, many studies have been conducted in marine, terrestrial, and human gut systems owing to growing interests in VB_12_ in different ecosystems. These studies focused on the quantification and classification of genes encoding cobalamin biosynthesis proteins, identification of multiple cobalamin transporters, and the combined effects of cobalamin and several other factors on the microbiota and environment (3, 5, 24, 27). Analysis of terrestrial metagenomes indicated that less than 10% of the total microbial community had a complete VB_12_ biosynthesis pathway (11), while most organisms require cobalamin to complete a series of necessary biological processes. Surprisingly, more than 98% of the total cobalamin demand of human gut microbiota requires exogenous supply to maintain the structure and function of gut microbial communities (3, 4). As elaborate processes are required to produce this cofactor, it has been shown that only a subset of bacteria and archaea can complete this process, emerging the “corrinoid economy” within the gut (1). Therefore, VB_12_ has been considered a critical limiting factor that affects the growth of other organisms and their interactions (2). It has been speculated that the producer of cobalamin serves a “keystone function” (11), akin to the notion of keystone species, and may greatly affect the biogeochemical functioning of microbial communities in various ecosystems. In addition, the VB_12_ biosynthesis capabilities could affect the behavioral characteristics of organisms, as well as physiological and developmental functions, such as enhancing the predatory feeding behavior of *Pristionchus* nematodes, resulting in surplus killing (28), and affecting the volume, growth rate, and life span of the organism (5). Most importantly, some neuronal circuits may be directly or indirectly affected by VB_12_ (5). Therefore, exploring the cobalamin biosynthesis processes and the taxonomic composition of the cobalamin producers is of great significance to clarify the stability and versatility of natural ecosystems and gut microbial ecology.

The developed VB_12_Path is a useful tool for studying cobalamin biosynthesis pathways because it provides accurate functional and taxonomic profiles of the pathway gene (sub)family of microorganisms in complex environments. Accuracy is a central issue for functional gene databases like VB_12_Path, which ensures high accuracy for subsequently generated functional profiles. The accuracy of VB_12_Path is guaranteed at two different levels. The first one is at the gene family level. An accurate and complete gene catalog in the database can usually be used as a reliable reference to help compile large-scale shotgun sequencing tasks (29, 30). VB_12_Path covers 60 key gene families involved in cobalamin biosynthesis, allowing researchers to obtain comprehensive functional gene profiles for microbial communities responsible for cobalamin biosynthesis in complex environments. In addition to commonly known gene families, VB_12_Path also covered a series of gene families that are currently missing in public databases, such as *ubiB* for DMB synthesis, *pduO* for cobinamide adenylation (31, 32), and *cobR* for reduction of cob(II)byrinic acid a,c-diamide (33). In addition, VB_12_Path also distinguished gene families that share high sequence identity and are usually merged as one orthologous group in public databases, although these gene families play different functions, such as *cobC*, *cobS*, *cobT*, and *cobU*.

The second level of accuracy lies at the sequence level. Accurate functional and taxonomic profiling relies not only on accurate definition of gene families but also on accurate recruitment of sequences for these gene families. Such high accuracy in sequence databases is of critical importance for reliable postprocessing metagenomic data analysis, such as inferring metabolic potential and characterizing functional and taxonomic compositions (34, 35). To ensure the accuracy of retrieved sequences, multiple actions were taken by VB_12_Path. First, seed sequences were downloaded from Swiss-Prot database, in which all sequences were manually curated by experts in the field (36). All seed sequences were subject to a second manual checking before downloading. Second, sequences downloaded from TrEMBL database were first checked against seed sequences based on sequence identity and then assigned to corresponding gene families based on majority rules if homologs exist. Third, sequences from public orthologous databases were also recruited, which were double checked based on sequence identity and majority rules. Meanwhile, their corresponding homologous orthologous groups were also collected to eliminate false-positive assignments in metagenomic analyses. In addition, we also merged NCBI RefSeq database with VB_12_Path. All these steps guaranteed a comprehensive and accurate coverage of high-quality sequences in VB_12_Path, providing accurate functional gene assignment for shotgun metagenome sequencing analysis.

In the metagenomic era, a large amount of data is generated with the development of next-generation sequencing technology (17), posing huge challenges in processing and analyzing such large data sets. To expedite this procedure, development of customized databases has emerged (21, 37, 38). As an essential micronutrient required by almost all organisms in both natural and artificial ecosystems, cobalamin is one of the most frequently analyzed cofactors in the environments, and its biosynthesis pathways have attracted more and more attention (5, 11, 23). By referencing the framework for knowledge-based database construction in NCycDB (21), VB_12_Path was developed to expedite data mining of gene families responsible for VB_12_ biosynthesis in complex metagenomes. VB_12_Path not only inherited critical characteristics of knowledge-based small databases such as comprehensive coverage of gene families, high accuracy, fast database searching, and low false-positive assignments but also provided solutions for both functional and taxonomic profiling of microbial communities involved in VB_12_ biosynthesis processes, thereby achieving in-depth insights into microbial VB_12_ biosynthesis pathways at both functional gene and taxonomic levels.

We applied VB_12_Path to identify and explore VB_12_ biosynthesis gene families in the ocean and human intestinal environments. The taxonomic profiles are consistent with previous reports that *Cyanobacteria* (39–41), the most representative producer of pseudocobalamin, is the predominant group of cobalamin producers in ocean environment. *Proteobacteria* presents as the second most abundant group (23). Studies have shown that the production of pseudocobalamin is likely an ancient relic (27), but the reason for the production and replacement of cobalamin is unknown. Some researchers have suggested that due to impaired DMB synthesis, adenine is used to replace DMB in certain organisms to produce pseudocobalamin (42). Although most sequences are assigned to *Cyanobacteria*, considering the cobalamin synthesis potential, in addition to the genomic potential, cobalamin cell quotas, abundance, and environmental factors must also be considered (27). At the class level, microbial genera belonging to *Gammaproteobacteria* and *Alphaproteobacteria* appear to be the most abundant groups, consistent with previous inferences (8, 23), as these groups contain many genes involved in corrin biosynthesis and DMB synthesis and activation. In the human intestinal environment, dramatically different microbial functional gene and taxonomic compositions of VB_12_ synthesis pathways were observed compared to those observed in the ocean environment. Such distinction could be due to the huge differences in environmental conditions between ocean and human intestine. Interestingly, for both ocean and human intestine, it could be observed that the compositions of functional gene families were more similar among different samples than were those of taxonomic groups, even at the phylum level. This is generally consistent with a previous study in human gut that found that different individuals hold a core microbiome at the gene level rather than at the lineage level (43). Similar patterns were also observed in the TARA Oceans microbiome study (44). In addition, according to the taxonomic profile, it was noticed that fewer than 5% of the sequences were assigned to archaea. This may be partly due to the reason that a relatively small number of archaea were sequenced, but another reason may be the fact that bacteria dominate VB_12_ biosynthesis processes in ocean and human intestine environments.

Taken together, VB_12_Path is a curated functional gene database that can be used as a toolkit for metagenomic profiling of VB_12_ biosynthesis gene families in various complex environments. VB_12_Path integrates multiple homology databases with manual verification to improve the coverage and accuracy of gene families and sequences. By applying VB_12_Path to marine and human intestine metagenomic samples, microbial gene (sub)families responsible for VB_12_ biosynthesis in ocean and human intestines were characterized. The profiles show that, while the taxonomic groups involved in VB_12_ biosynthesis vary highly, VB_12_ synthetic gene families are highly consistent among different samples. In conclusion, VB_12_Path provides a platform for shotgun metagenome data analysis, revealing accurate taxonomic and functional profiles for VB_12_ biosynthesis gene families of microbial communities from different environments.

## MATERIALS AND METHODS

### Framework development.

**FIG 5:**
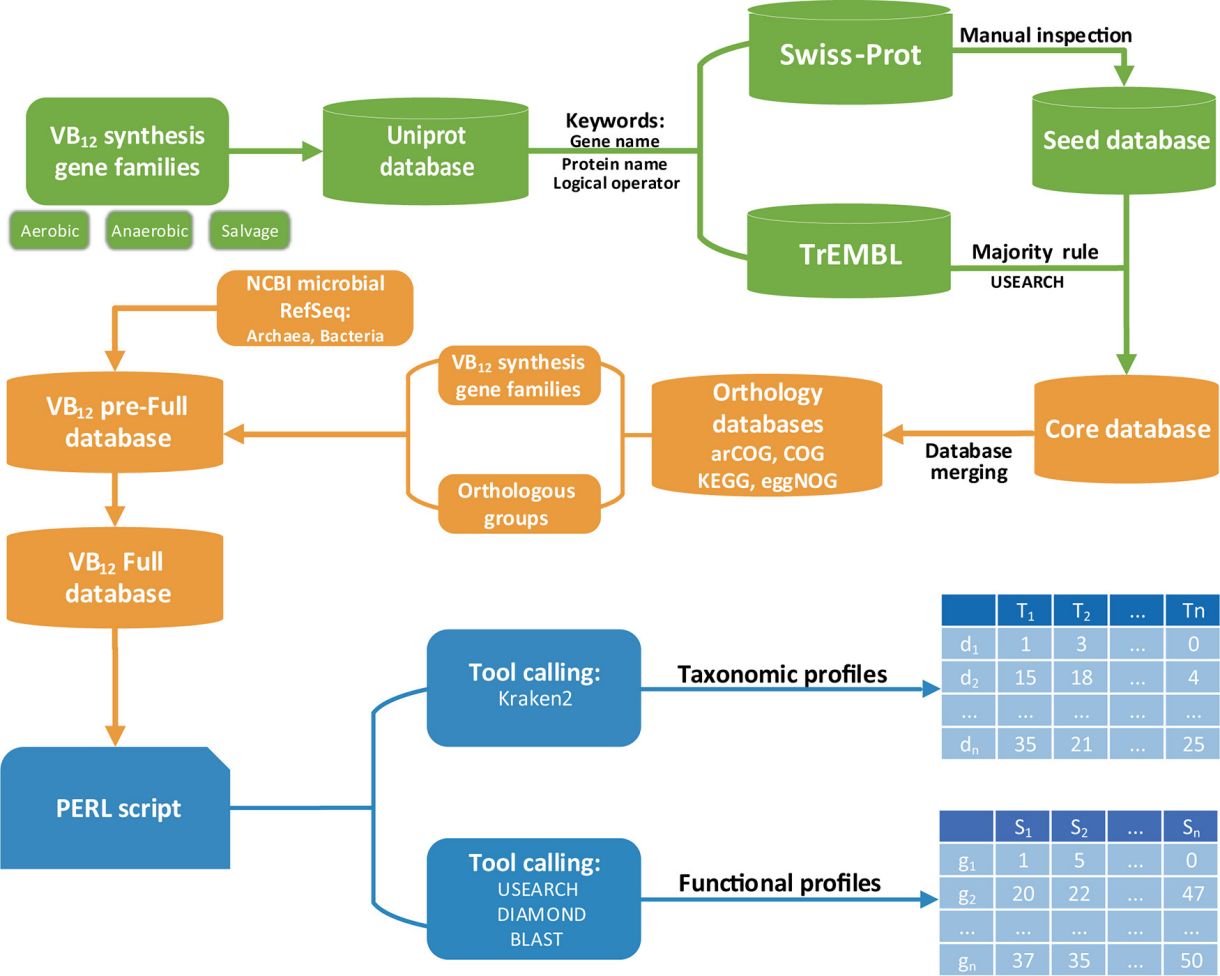
Workflow summary for constructing VB_12_Path. Multiple steps were included in the framework, including seed database construction, core database construction, database merging, and full database construction. A set of PERL scripts are also provided to generate taxonomic and functional gene profiles from shotgun metagenomes.

Based on the framework previously shown in NCycDB (21), a pipeline was developed for VB_12_Path with moderate modifications (Fig. 5). Overall, the pipeline encompassed four major steps, including seed database construction, core database construction, full database construction, and metagenomic profiling.

### Seed database construction.

A seed database was first constructed for VB_12_Path. The seed database needs to contain highly accurate reference sequences for each gene family involved in VB_12_ biosynthesis, thereby ensuring the accuracy of VB_12_Path at the very beginning. Here, curated reference sequences from the Swiss-Prot database (45) were retrieved. To do so, we first determined the specific processes of VB_12_ biosynthesis by referring to multiple literatures (11, 23, 31) and the VB_12_ synthesis pathways in KEGG database (18). Key gene families involved in the VB_12_ biosynthesis pathways were then identified. For each gene family, keywords were determined based on their gene name and corresponding protein name (Table S1). The keywords were further refined by manually checking the searching results. In some cases, logical operators were used in order to include wanted or exclude unwanted sequences so that the gene family was most appropriately described (e.g., gene:*hemA* name:”glutamyl-trna reductase” NOT name:”multifunctional siroheme biosynthesis protein *hemA*”). Notably, some genes may have multiple protein names and play different roles in different processes in VB_12_ synthetic pathways. One such example is *cobC*, which is the gene name for threonine-phosphate decarboxylase and adenosylcobalamin phosphatase. Of them, threonine-phosphate decarboxylase plays a role in the direct attachment of (*R*)-1-amino-2-propanol to the corrinoid ring, while the adenosylcobalamin phosphatase catalyzes dephosphorylation of adenosylcobalamin (31). The retrieved sequences from Swiss-Prot database were then manually inspected to ensure their accuracy. Verified sequences were downloaded and stored in the seed database.

### Core database construction.

In addition to Swiss-Prot, sequences were also collected from the TrEMBL database, forming the core database. Sequences belonging to targeted gene families were downloaded from the TrEMBL database using the refined keywords. Since tens of thousands of sequences could be retrieved, these sequences were briefly inspected for their accuracy. For each gene family, these less-accurate sequences downloaded from TrEMBL database were then searched against their seed sequences by USEARCH (46) at a global identity cutoff of 30%. If homologs were found among gene families (e.g., *cysG* and *cobA*) involved in VB_12_ biosynthesis pathways, seed sequences of all homologous gene families were used in the USEARCH procedure. Sequences retrieved from TrEMBL database were assigned to the gene family with best hit.

### Full database construction.

In order to comprehend VB_12_Path and minimize false-positive effects due to “small database issue,” we also integrated multiple public databases into VB_12_Path. In addition to integrating targeted gene families in public orthology databases, we also identified and included homologous gene families. To do so, public orthology databases, including arCOG (Archaeal Clusters of Orthologous Genes) (47), COG (20), KEGG (18), and eggNOG (19), were searched against the core database. Orthologous groups belonging to targeted gene families as well as homologous orthologous groups were identified based on semimanual inspection efforts. Sequences were extracted and merged with known targeted gene families or included as homologous groups, forming a “pre-full” database. After that, both archaeal and bacterial RefSeq databases in NCBI were downloaded and searched against the pre-full and public orthology databases. Sequences best targeting VB_12_ gene families and their homologous groups were then respectively extracted and merged, forming the full database. The CD-HIT program (48) was then used to generate a set of nonredundant representative sequences at 100% identity cutoff.

### Metagenomic profiling of VB_12_ gene families.

Included in VB_12_Path, we also provided a set of PERL scripts for database searching and metagenomic profiling of VB_12_ gene families. Both functional gene and taxonomic profiles for targeted VB_12_ gene families were generated. For functional profiling, database searching tools including DIAMOND (49), USEARCH (46), and BLAST (50) were supported. Other database searching software can also be integrated easily. For taxonomic profiling, sequences targeting VB_12_ gene families were extracted based on database searching report files by the seqtk program (51). The tool Kraken2 (52) was then called for taxonomic assignment of these extracted sequences. Taxonomic profiles at different taxonomic levels were then generated. Further statistical analyses could be carried out for both functional and taxonomic profiles at users’ own choices.

### Case study procedures.

In order to evaluate the performance of VB_12_Path in profiling VB_12_ gene families and their taxonomic information in complex metagenomes, we collected 25 representative shotgun metagenome data sets, among which 10 came from the Human Microbiome Project and 15 came from the TARA Oceans expedition. The human gut metagenomes were downloaded from https://www.hmpdacc.org. The TARA Oceans metagenome data sets were downloaded from http://ocean-microbiome.embl.de/companion.html. Forward and reverse reads were merged into longer sequences by the PEAR program (version 0.9.6, -q 30) (53). The merged sequences were then searched against VB_12_Path using the DIAMOND program (-k 1 -e 0.0001 -id 0.3) (49). Functional profiles were generated based on the DIAMOND output files. Metagenomic sequences mapped to VB_12_ biosynthesis gene families were extracted by the seqtk program (version 1.3) (51). The Kraken2 program (52) was employed for taxonomic assignment for the extracted reads. A standard database was built for Kraken2 which covered complete genomes in RefSeq for the bacterial, archaeal, and viral domains, along with the human genome and a collection of known vectors (UniVec_Core). Taxonomic profiles of VB_12_ biosynthesis communities were generated at different taxonomic levels. The generated functional gene and taxonomic profiles were then used for further statistical analyses.

### Database sources.

Keywords searching and sequence retrieval for seed and core databases were carried out at the UniProt website (https://www.uniprot.org/). The following orthologous databases were downloaded and integrated in this study, including arCOG downloaded from https://ftp.ncbi.nih.gov/pub/wolf/ COGs, COG downloaded from https://ftp.ncbi.nlm.nih.gov/pub/COG/COG2014/data/prot2003-2014.fa.gz, KEGG downloaded from http://www.genome.jp/kegg/, and eggNOG downloaded from http://eggnogdb.embl.de/. Archaeal and bacterial nonredundant RefSeq databases were respectively downloaded from http://ftp.ncbi.nlm.nih.gov/refseq/release/archaea/ and http://ftp.ncbi.nlm.nih.gov/refseq/release/bacteria/.

### Data availability.

VB_12_Path is available at https://github.com/qichao1984/VB12Path.

## References

[1] Sonnenburg ED, Sonnenburg JL. 2014. Gut microbes take their vitamins. Cell Host Microbe 15:5–6. doi:10.1016/j.chom.2013.12.011. 24439893 PMC4111938

[2] King AL, Sañudo-Wilhelmy SA, Leblanc K, Hutchins DA, Fu F. 2011. CO_2_ and vitamin B_12_ interactions determine bioactive trace metal requirements of a subarctic Pacific diatom. ISME J 5:1388–1396. doi:10.1038/ismej.2010.211. 21248860 PMC3146264

[3] Degnan PH, Barry NA, Mok KC, Taga ME, Goodman AL. 2014. Human gut microbes use multiple transporters to distinguish vitamin B_12_ analogs and compete in the gut. Cell Host Microbe 15:47–57. doi:10.1016/j.chom.2013.12.007. 24439897 PMC3923405

[4] Degnan PH, Taga ME, Goodman AL. 2014. Vitamin B_12_ as a modulator of gut microbial ecology. Cell Metabolism 20:769–778. doi:10.1016/j.cmet.2014.10.002. 25440056 PMC4260394

[5] Akduman N, Lightfoot JW, Rseler W, Witte H, Lo W-S, Rödelsperger C, Sommer RJ. 2020. Bacterial vitamin B_12_ production enhances nematode predatory behavior. ISME J 14:1494–1507. doi:10.1038/s41396-020-0626-2. 32152389 PMC7242318

[6] Warren MJ, Raux E, Schubert HL, Escalante-Semerena JC. 2002. The biosynthesis of adenosylcobalamin (vitamin B_12_). Nat Prod Rep 19:390–412. doi:10.1039/b108967f. 12195810

[7] Croft MT, Lawrence AD, Raux-Deery E, Warren MJ, Smith A. 2005. Algae acquire vitamin B_12_ through a symbiotic relationship with bacteria. Nature 438:90–93. doi:10.1038/nature04056. 16267554

[8] Sañudo-Wilhelmy SA, Gomez-Consarnau L, Suffridge C, Webb EA. 2014. The role of B vitamins in marine biogeochemistry. Annu Rev of Marine Science 6:339–367. doi:10.1146/annurev-marine-120710-100912. 24050603

[9] Ortiz-Guerrero JM, Polanco MC, Murillo FJ, Padmanabhan S, Elías-Arnanz M. 2011. Light-dependent gene regulation by a coenzyme B_12_-based photoreceptor. Proc Natl Acad Sci U S A 108:7565–7570. doi:10.1073/pnas.1018972108. 21502508 PMC3088613

[10] Roth J, Lawrence J, Bobik T. 1996. Cobalamin (coenzyme B_12_): synthesis and biological significance. Annu Rev of Microbiology 50:137–181. doi:10.1146/annurev.micro.50.1.137. 8905078

[11] Lu X, Heal KR, Ingalls AE, Doxey AC, Neufeld JD. 2020. Metagenomic and chemical characterization of soil cobalamin production. ISME J 14:53–66. doi:10.1038/s41396-019-0502-0. 31492962 PMC6908642

[12] Heal KR, Carlson LT, Devol AH, Armbrust EV, Moffett JW, Stahl DA, Ingalls AE. 2014. Determination of four forms of vitamin B_12_ and other B vitamins in seawater by liquid chromatography/tandem mass spectrometry. Rapid Commun Mass Spectrom 28:2398–2404. doi:10.1002/rcm.7040. 25303468

[13] Fang H, Li D, Kang J, Jiang P, Sun J, Zhang D. 2018. Metabolic engineering of Escherichia coli for de novo biosynthesis of vitamin B_12_. Nat Commun 9:4917. doi:10.1038/s41467-018-07412-6. 30464241 PMC6249242

[14] Warren MJ, Escalante-Semerena J. 2008. Biosynthesis and use of cobalamin (B_12_). Ecosal Plus 3. doi:10.1128/ecosalplus.3.6.3.8. 26443728

[15] Woodcock SC, Raux E, Levillayer F, Thermes C, Rambach A, Warren MJ. 1998. Effect of mutations in the transmethylase and dehydrogenase/chelatase domains of sirohaem synthase (CysG) on sirohaem and cobalamin biosynthesis. Biochemistry 330:121–129. doi:10.1042/bj3300121. PMC1219117 9461500

[16] Rodionov DA, Vitreschak AG, Mironov AA, Gelfand MS. 2003. Comparative genomics of the vitamin B_12_ metabolism and regulation in prokaryotes. J Biological Chemistry 278:41148–41159. doi:10.1074/jbc.M305837200. 12869542

[17] Scholz MB, Lo CC, Chain PS. 2012. Next generation sequencing and bioinformatic bottlenecks: the current state of metagenomic data analysis. Curr Opin Biotechnol 23:9–15. doi:10.1016/j.copbio.2011.11.013. 22154470

[18] Kanehisa M, Sato Y, Kawashima M, Furumichi M, Tanabe M. 2016. KEGG as a reference resource for gene and protein annotation. Nucleic Acids Res 44:D457–D462. doi:10.1093/nar/gkv1070. 26476454 PMC4702792

[19] Huerta-Cepas J, Szklarczyk D, Heller D, Hern´Andez-Plaza A, Forslund SK, Cook H, Mende DR, Letunic I, Rattei T, Jensen LJ, Mering C, Bork P. 2019. eggNOG 5.0: a hierarchical, functionally and phylogenetically annotated orthology resource based on 5090 organisms and 2502 viruses. Nucleic Acids Res 47:D309–D314. doi:10.1093/nar/gky1085. 30418610 PMC6324079

[20] Galperin MY, Makarova KS, Wolf YI, Koonin EV. 2015. Expanded microbial genome coverage and improved protein family annotation in the COG database. Nucleic Acids Res 43:D261–D269. doi:10.1093/nar/gku1223. 25428365 PMC4383993

[21] Tu Q, Lin L, Cheng L, Deng Y, He Z. 2019. NCycDB: a curated integrative database for fast and accurate metagenomic profiling of nitrogen cycling genes. Bioinformatics 35:1040–1048. doi:10.1093/bioinformatics/bty741. 30165481

[22] Stubbe J. 1994. Binding site revealed of nature’s most beautiful cofactor. Science 266:1663–1664. doi:10.1126/science.7992049. 7992049

[23] Doxey AC, Kurtz DA, Lynch MD, Sauder LA, Neufeld JD. 2015. Aquatic metagenomes implicate Thaumarchaeota in global cobalamin production. ISME J 9:461–471. doi:10.1038/ismej.2014.142. 25126756 PMC4303638

[24] Bertrand EM, McCrow JP, Moustafa A, Zheng H, McQuaid JB, Delmont TO, Post AF, Sipler RE, Spackeen JL, Xu K, Bronk DA, Hutchins DA, Allen AE. 2015. Phytoplankton-bacterial interactions mediate micronutrient colimitation at the coastal Antarctic sea ice edge. Proc Natl Acad Sci U S A 112:9938–9943. doi:10.1073/pnas.1501615112. 26221022 PMC4538660

[25] Giedyk M, Goliszewska K, Gryko D. 2015. Vitamin B_12_ catalysed reactions. Chem Soc Rev 44:3391–3404. doi:10.1039/C5CS00165J. 25945462

[26] Schulz R-J. 2007. Homocysteine as a biomarker for cognitive dysfunction in the elderly. Curr Op Clin Nutr Metab Care 10:718–723. doi:10.1097/MCO.0b013e3282f0cfe3. 18089953

[27] Heal KR, Qin W, Ribalet F, Bertagnolli AD, Coyote-Maestas W, Hmelo LR, Moffett JW, Devol AH, Armbrust EV, Stahl DA, Ingalls AE. 2017. Two distinct pools of B_12_ analogs reveal community interdependencies in the ocean. Proc Natl Acad Sci U S A 114:364–369. doi:10.1073/pnas.1608462114. 28028206 PMC5240700

[28] Zimmermann B, Sand H, Wabakken P, Liberg O, Andreassen HPJ. 2015. Predator-dependent functional response in wolves: from food limitation to surplus killing. J Anim Ecol 84:102–112. doi:10.1111/1365-2656.12280. 25109601

[29] Li J, Jia H, Cai X, Zhong H, Feng Q, Sunagawa S, Arumugam M, Kultima JR, Prifti E, Nielsen T, Juncker AS, Manichanh C, Chen B, Zhang W, Levenez F, Wang J, Xu X, Xiao L, Liang S, Zhang D, Zhang Z, Chen W, Zhao H, Al-Aama JY, Edris S, Yang H, Wang J, Hansen T, Nielsen HB, Brunak S, Kristiansen K, Guarner F, Pedersen O, Dore J, Ehrlich SD, Meta HITC, Bork P, Wang J, Meta HITC. 2014. An integrated catalog of reference genes in the human gut microbiome. Nat Biotechnol 32:834–841. doi:10.1038/nbt.2942. 24997786

[30] Xiao L, Feng Q, Liang S, Sonne SB, Xia Z, Qiu X, Li X, Long H, Zhang J, Zhang D, Liu C, Fang Z, Chou J, Glanville J, Hao Q, Kotowska D, Colding C, Licht TR, Wu D, Yu J, Sung JJ, Liang Q, Li J, Jia H, Lan Z, Tremaroli V, Dworzynski P, Nielsen HB, Backhed F, Dore J, Le Chatelier E, Ehrlich SD, Lin JC, Arumugam M, Wang J, Madsen L, Kristiansen K. 2015. A catalog of the mouse gut metagenome. Nat Biotechnol 33:1103–1108. doi:10.1038/nbt.3353. 26414350

[31] Fang H, Kang J, Zhang D. 2017. Microbial production of vitamin B_12_: a review and future perspectives. Microb Cell Fact 16:15. doi:10.1186/s12934-017-0631-y. 28137297 PMC5282855

[32] Moore TC, Newmister SA, Rayment I, Escalante-Semerena CJ. 2012. Structural insights into the mechanism of four-coordinate Cob(II)alamin formation in the active site of the Salmonella enterica ATP:Co(I)rrinoid adenosyltransferase enzyme: critical role of residues Phe91 and Trp93. Biochemistry 51:9647–9657. doi:10.1021/bi301378d. 23148601 PMC3567240

[33] Scott AI, Roessner CA. 2002. Biosynthesis of cobalamin (vitamin B_12_). Biochemical Soc Trans 30:613–620. doi:10.1042/bst0300613. 12196148

[34] Huttenhower C, Gevers D, Knight R, Abubucker S, H Badger J. 2012. Structure, function and diversity of the healthy human microbiome. Nature 486:207–214. doi:10.1038/nature11234. 22699609 PMC3564958

[35] Quince C, Walker AW, Simpson JT, Loman NJ, Segata N. 2017. Shotgun metagenomics, from sampling to analysis. Nat Biotechnol 35:833–844. doi:10.1038/nbt.3935. 28898207

[36] UniProt Consortium. 2019. UniProt: a worldwide hub of protein knowledge. Nucleic Acids Res 47:D506–D515. doi:10.1093/nar/gky1049. 30395287 PMC6323992

[37] Emmanuel P, Dm M, Jenni H, Neslihan T, Regina L, Jill D, Rachel M, Md D, Ari J, Ts G, Elizabeth H, Konstantinos M, Jj K. 2014. FOAM (functional ontology assignments for metagenomes): a hidden Markov model (HMM) database with environmental focus. Nucleic Acids Res 42:e145. doi:10.1093/nar/gku702. 25260589 PMC4231724

[38] Dunivin TK, Yeh SY, Shade A. 2019. A global survey of arsenic-related genes in soil microbiomes. BMC Biology 17:45. doi:10.1186/s12915-019-0661-5. 31146755 PMC6543643

[39] Helliwell K, Lawrence A, Holzer A, Kudahl U, Sasso S, Krutler B, Scanlan D, Warren M, Smith A. 2016. Cyanobacteria and eukaryotic algae use different chemical variants of vitamin B_12_. Curr Biol 26:999–1008. doi:10.1016/j.cub.2016.02.041. 27040778 PMC4850488

[40] Tanioka Y, Miyamoto E, Yabuta Y, Ohnishi K, Fujita T, Yamaji R, Misono H, Shigeoka S, Nakano Y, Inui H. 2010. Methyladeninylcobamide functions as the cofactor of methionine synthase in a Cyanobacterium, Spirulina platensis NIES-39. FEBS Lett 584:3223–3226. doi:10.1016/j.febslet.2010.06.013. 20558164

[41] Tanioka Y, Yabuta Y, Yamaji R, Shigeoka S, Nakano Y, Watanabe F, Inui H. 2009. Occurrence of pseudovitamin B_12_ and its possible function as the cofactor of cobalamin-dependent methionine synthase in a cyanobacterium Synechocystis sp. PCC6803. J Nutr Sci Vitaminol (Tokyo) 55:518–521. doi:10.3177/jnsv.55.518. 20086323

[42] Cheong CG, Escalante-Semerena JC, Rayment I. 2001. Structural investigation of the biosynthesis of alternative lower ligands for cobamides by nicotinate mononucleotide: 5,6-dimethylbenzimidazole phosphoribosyltransferase from Salmonella enterica. J Biol Chem 276:37612–37620. doi:10.1074/jbc.M105390200. 11441022

[43] Turnbaugh PJ, Hamady M, Yatsunenko T, Cantarel BL, Duncan A, Ley RE, Sogin ML, Jones WJ, Roe BA, Affourtit JP, Egholm M, Henrissat B, Heath AC, Knight R, Gordon JI. 2009. A core gut microbiome in obese and lean twins. Nature 457:480–484. doi:10.1038/nature07540. 19043404 PMC2677729

[44] Sunagawa S, Coelho LP, Chaffron S, Kultima JR, Labadie K, Salazar G, Djahanschiri B, Zeller G, Mende DR, Alberti A, Cornejo-Castillo FM, Costea PI, Cruaud C, d’Ovidio F, Engelen S, Ferrera I, Gasol JM, Guidi L, Hildebrand F, Kokoszka F, Lepoivre C, Lima-Mendez G, Poulain J, Poulos BT, Royo-Llonch M, Sarmento H, Vieira-Silva S, Dimier C, Picheral M, Searson S, Kandels-Lewis S, Bowler C, de Vargas C, Gorsky G, Grimsley N, Hingamp P, Iudicone D, Jaillon O, Not F, Ogata H, Pesant S, Speich S, Stemmann L, Sullivan MB, Weissenbach J, Wincker P, Karsenti E, Raes J, Acinas SG, Bork P, Tara Oceans coordinators, et al. 2015. Structure and function of the global ocean microbiome. Science 348:1261359. doi:10.1126/science.1261359. 25999513

[45] Boeckmann B, Bairoch A, Apweiler R, Blatter MC, Estreicher A, Gasteiger E, Martin MJ, Michoud K, O'Donovan C, Phan I, Pilbout S, Schneider M. 2003. The SWISS-PROT protein knowledgebase and its supplement TrEMBL in 2003. Nucleic Acids Res 31:365–370. doi:10.1093/nar/gkg095. 12520024 PMC165542

[46] Edgar RC. 2010. Search and clustering orders of magnitude faster than BLAST. Bioinformatics 26:2460–2461. doi:10.1093/bioinformatics/btq461. 20709691

[47] Makarova K, Wolf Y, Koonin E. 2015. Archaeal clusters of orthologous genes (arCOGs): An update and application for analysis of shared features between Thermococcales, Methanococcales, and Methanobacteriales. Life 5:818–840. doi:10.3390/life5010818. 25764277 PMC4390880

[48] Li W, Godzik A. 2006. Cd-hit: a fast program for clustering and comparing large sets of protein or nucleotide sequences. Bioinformatics 22:1658–1659. doi:10.1093/bioinformatics/btl158. 16731699

[49] Buchfink B, Xie C, Huson DH. 2015. Fast and sensitive protein alignment using DIAMOND. Nat Methods 12:59–60. doi:10.1038/nmeth.3176. 25402007

[50] Altschul SF, Gish W, Miller W, Myers EW, Lipman DJ. 1990. Basic local alignment search tool. Molecular Biology 215:403–410. doi:10.1016/S0022-2836(05)80360-2. 2231712

[51] seqtk, toolkit for processing sequences in FASTA/Q formats. https://github.com/lh3/seqtk.

[52] Wood DE, Lu J, Langmead B. 2019. Improved metagenomic analysis with Kraken 2. Genome Biol 20:257. doi:10.1186/s13059-019-1891-0. 31779668 PMC6883579

[53] Zhang J, Kobert K, Flouri T, Stamatakis A. 2014. PEAR: a fast and accurate Illumina paired-end reAd mergeR. Bioinformatics 30:614–620. doi:10.1093/bioinformatics/btt593. 24142950 PMC3933873

