# Supplementary material for: VB12Path for Accurate Metagenomic Profiling of Microbially Driven Cobalamin Synthesis Pathways
Source: mSystems. 2021 Jun 1;6(3):e00497-21. doi: 10.1128/mSystems.00497-21 (PMC8269236; doi:10.1128/mSystems.00497-21)
Supplement: TABLE S1 [file msystems.00497-21-st001.docx]

| Gene | Protein | Key words |
| --- | --- | --- |
| hemA | Glutamyl-tRNA reductase | gene:hema name:"glutamyl-trna reductase" NOT name:"probable multifunctional siroheme biosynthesis protein hema" AND reviewed:yes gene:hema name:"glutamyl-trna reductase" AND reviewed:no |
| hemL | Glutamate-1-semialdehyde 2,1-aminomutase | gene:heml name:"glutamate-1-semialdehyde 2,1-aminomutase" AND reviewed:yes gene:heml name:"glutamate-1-semialdehyde 2,1-aminomutase" NOT name:"multifunctional fusion protein" AND reviewed:no |
| gltX | Glutamate--tRNA ligase | gene:gltx (name:"glutamate--trna ligase" NOT name:"glutamylglutaminyl-trna synthetase") AND reviewed:yes gene:gltx (name:"glutamate--trna ligase" NOT name:"multifunctional fusion protein") AND reviewed:no |
| hemB | Delta-aminolevulinic acid dehydratase | gene:hemb name:"delta-aminolevulinic acid dehydratase" AND reviewed:yes gene:hemb name:"delta-aminolevulinic acid dehydratase" AND reviewed:no |
| hemC | Porphobilinogen deaminase | gene:hemc name:"porphobilinogen deaminase" AND reviewed:yes (gene:hemc NOT gene:hema) name:"porphobilinogen deaminase" AND reviewed:no |
| hemD | Uroporphyrinogen-III synthase | (gene:hemd NOT gene:uros) (name:"uroporphyrinogen-III synthase" NOT name:"porphyrin biosynthesis protein hemd") AND reviewed:yes (gene:hemd NOT gene:coba) name:"uroporphyrinogen-III synthase" AND reviewed:no |
| cysG | Siroheme synthase | gene:cysg name:"siroheme synthase" AND reviewed:yes (gene:cysg NOT gene:coba) NOT gene:cysf name:"siroheme synthase" AND reviewed:no |
| cobA | uroporphyrinogen-III c-methyltransferase | gene:coba NOT gene:cysg name:"uroporphyrinogen- III c-methyltransferase" AND reviewed:yes  gene:coba name:"uroporphyrinogen- III c-methyltransferase" AND reviewed:no |
| cobB | Hydrogenobyrinate a,c-diamide synthase | gene:cobb name:"hydrogenobyrinate a,c-diamide synthase" AND reviewed:yes gene:cobb name:"hydrogenobyrinate a,c-diamide synthase" AND reviewed:no |
| cobC-beta | Threonine-phosphate decarboxylase | gene:cobc name:"threonine-phosphate decarboxylase" AND reviewed:yes gene:cobc name:"threonine-phosphate decarboxylase" AND reviewed:no |
| cobC-ado | Adenosylcobalamin/alpha-ribazole phosphatase | gene:cobc name:"adenosylcobalamin alpha-ribazole phosphatase" AND reviewed:yes gene:cobc name:"adenosylcobalamin alpha-ribazole phosphatase" AND reviewed:no |
| cobD | Threonine-phosphate decarboxylase | gene:cobd name:"threonine-phosphate decarboxylase" AND reviewed:yes gene:cobd name:"threonine-phosphate decarboxylase" AND reviewed:no |
| cobF | Precorrin-6A synthase | gene:cobf name:"precorrin-6A synthase" AND reviewed:yes gene:cobf NOT gene:cbid name:"precorrin-6A synthase" AND reviewed:no |
| cobG | Precorrin-3B synthase | gene:cobg name:"precorrin-3B synthase" AND reviewed:yes gene:cobg name:"precorrin-3B synthase" AND reviewed:no |
| cobH | Precorrin-8X methylmutase | gene:cobh name:"precorrin-8X methylmutase" AND reviewed:yes gene:cobh (name:"precorrin-8X methylmutase" NOT name:"precorrin-8X methylmutase or isomerase") AND reviewed:no |
| cobI | Precorrin-2 C(20)-methyltransferase | (gene:cobi NOT gene:cobij) name:"precorrin-2 C(20) -methyltransferase" AND reviewed:yes (gene:cobi NOT gene:cbil) (name:"precorrin-2 C(20) -methyltransferase" NOT name:"cobalt- precorrin-2 C(20)-methyltransferase") AND reviewed:no |
| cobJ | Precorrin-3B C(17)-methyltransferase | gene:cobj name:"precorrin-3B C(17) -methyltransferase" AND reviewed:yes gene:cobj (name:"precorrin-3B C(17) -methyltransferase" NOT name:cobalt) AND reviewed:no |
| cobK | Precorrin-6A reductase | gene:cobk NOT gene:cbij name:"precorrin-6A reductase" AND reviewed:yes gene:cobk NOT gene:cbij (name:"precorrin-6A reductase" NOT name:'cobalt') AND reviewed:no |
| cobL | Precorrin-6Y C(5,15)-methyltransferase | gene:cobl name:"precorrin-6Y C(5,15)-methyltransferase" AND reviewed:yes gene:cobl name:"precorrin-6Y C(5,15)-methyltransferase" AND reviewed:no |
| cobM | Precorrin-4 C(11)-methyltransferase | gene:cobm name:"precorrin-4 C(11) -methyltransferase" AND reviewed:yes gene:cobm NOT gene:cbif name:"precorrin-4 C(11)-methyltransferase" AND reviewed:no |
| cobN | Aerobic cobaltochelatase subunit CobN | gene:cobn name:"aerobic cobaltochelatase subunit cobn" AND reviewed:yes gene:cobn name:"aerobic cobaltochelatase subunit cobn" AND reviewed:no |
| cobO | Corrinoid adenosyltransferase | gene:cobo name:"corrinoid adenosyltransferase" AND reviewed:yes gene:cobo (name:"corrinoid adenosyltransferase" NOT name:atp NOT name:"cob(I)alamin adenosyltransferase cobo") AND reviewed:no |
| cobP | Bifunctional adenosylcobalamin biosynthesis protein CobP | gene:cobp name:"bifunctional adenosylcobalamin biosynthesis protein cobp" AND reviewed:yes gene:cobp name:"bifunctional adenosylcobalamin biosynthesis protein" AND reviewed:no |
| cobQ | Cobyric acid synthase | gene:cobq name:"cobyric acid synthase" AND reviewed:yes gene:cobq (name:"cobyric acid synthase" NOT name:cobq) AND reviewed:no |
| cobR | Cob(II)yrinic acid a,c-diamide reductase | gene:cobr name:"cob(II)yrinic acid a,c-diamide reductase" AND reviewed:no |
| cobS-gdp | Aerobic cobaltochelatase subunit CobS | gene:cobs name:"aerobic cobaltochelatase subunit cobs" AND reviewed:yes gene:cobs name:"aerobic cobaltochelatase subunit cobs" NOT name:"atpase protein" AND reviewed:no |
| cobS-co | Adenosylcobinamide-GDP ribazoletransferase | gene:cobs name:"adenosylcobinamide-GDP ribazoletransferase" AND reviewed:yes (gene:cobs NOT gene:cobv) name:"adenosylcobinamide-GDP ribazoletransferase" NOT name:"multifunctional fusion protein" AND reviewed:no |
| cobT-co | Aerobic cobaltochelatase subunit CobT | gene:cobt name:"aerobic cobaltochelatase subunit cobt" AND reviewed:yes gene:cobt name:"aerobic cobaltochelatase subunit cobt" AND reviewed:no |
| cobT-alpha | Nicotinate-nucleotide--dimethylbenzimidazole phosphoribosyltransferase | gene:cobt name:"nicotinate-nucleotide--dimethylbenzimidazole phosphoribosyltransferase" AND reviewed:yes (gene:cobt NOT gene:blub) NOT gene:cobp NOT gene:cobu name:"nicotinate-nucleotide--dimethylbenzimidazole phosphoribosyltransferase" NOT name:"multifunctional fusion protein" AND reviewed:no |
| cobU-ade | Bifunctional adenosylcobalamin biosynthesis protein CobU | gene:cobu name:"bifunctional adenosylcobalamin biosynthesis protein cobu" AND reviewed:yes gene:cobu name:"bifunctional adenosylcobalamin biosynthesis protein cobu" AND reviewed:no |
| cobU-alpha | Nicotinate-nucleotide--dimethylbenzimidazole phosphoribosyltransferase | gene:cobu name:nicotinate-nucleotide--dimethylbenzimidazole phosphoribosyltransferase AND reviewed:yes gene:cobu name:"nicotinate-nucleotide--dimethylbenzimidazole phosphoribosyltransferase" AND reviewed:no |
| cobV | Adenosylcobinamide-GDP ribazoletransferase | gene:cobv NOT gene:cobs name:"adenosylcobinamide-GDP ribazoletransferase" AND reviewed:yes gene:cobv name:"adenosylcobinamide-GDP ribazoletransferase" AND reviewed:no |
| cobY | Adenosylcobinamide-phosphate guanylyltransferase | gene:coby name:"adenosylcobinamide-phosphate guanylyltransferase" AND reviewed:yes gene:coby NOT gene:cobu name:"adenosylcobinamide-phosphate guanylyltransferase" AND reviewed:no |
| pduX | L-threonine kinase | gene:pdux name:"L-threonine kinase" AND reviewed:yes gene:pdux name:"L-threonine kinase" AND reviewed:no |
| cbiA | Cobyrinate a,c-diamide synthase | gene:cbia name:"cobyrinate a,c-diamide synthase" AND reviewed:yes gene:cbia name:"cobyrinate a,c-diamide synthase" AND reviewed:no |
| cbiB | Cobalamin biosynthesis protein CbiB | gene:cbib name:"cobalamin biosynthesis protein cbib" AND reviewed:yes gene:cbib name:"cobalamin biosynthesis protein cbib" AND reviewed:no |
| cbiC | Cobalt-precorrin-8 methylmutase | gene:cbic name:"cobalt-precorrin-8 methylmutase" AND reviewed:yes gene:cbic name:"cobalt-precorrin-8 methylmutase" AND reviewed:no |
| cbiD | Cobalt-precorrin-5B C(1)-methyltransferase | gene:cbid name:"cobalt-precorrin-5B C(1) -methyltransferase" AND reviewed:yes gene:cbid NOT gene:yusv NOT gene:cobk NOT gene:cobh name:"cobalt-precorrin-5B C(1) -methyltransferase" NOT name:"multifunctional fusion protein" AND reviewed:no |
| cbiE | Cobalt-precorrin-7 C(5)-methyltransferase | gene:cbie name:"cobalt-precorrin-7 C(5) -methyltransferase" AND reviewed:yes gene:cbie name:"cobalt-precorrin-7 C(5) -methyltransferase" AND reviewed:no |
| cbiF | Cobalt-precorrin-4 C(11)-methyltransferase | gene:cbif name:"cobalt-precorrin-4 C(11) -methyltransferase" AND reviewed:yes gene:cbif name:"cobalt-precorrin-4 C(11) -methyltransferase" AND reviewed:no |
| cbiG | Cobalt-precorrin-5A hydrolase | gene:cbig name:"cobalt-precorrin-5A hydrolase" AND reviewed:yes gene:cbig name:"cobalt-precorrin-5A hydrolase" AND reviewed:no |
| cbiH | Cobalt-factor III C(17)-methyltransferase | gene:cbih name:"cobalt-factor III C(17)-methyltransferase" AND reviewed:yes gene:cbih NOT gene:cobj name:"cobalt-factor III C(17) -methyltransferase" AND reviewed:no |
| cbiJ | Cobalt-precorrin-6A reductase | gene:cbij name:"cobalt-precorrin-6A reductase" AND reviewed:yes gene:cbij name:"cobalt-precorrin-6A reductase" AND reviewed:no |
| cbiK | Sirohydrochlorin cobaltochelatase | gene:cbik name:"sirohydrochlorin cobaltochelatase" AND reviewed:yes gene:cbik name:"sirohydrochlorin cobaltochelatase" AND reviewed:no |
| cbiL | Cobalt-precorrin-2 C(20)-methyltransferase | gene:cbil name:"cobalt-precorrin-2 C(20) -methyltransferase" AND reviewed:yes gene:cbil name:"cobalt-precorrin-2 C(20) -methyltransferase" AND reviewed:no |
| cbiP | Cobyric acid synthase | gene:cbip NOT gene:cobq name:"cobyric acid synthase" AND reviewed:yes gene:cbip NOT gene:cbih name:"cobyric acid synthase" NOT name:"cobyric acid synthase cbip" AND reviewed:no |
| cbiT | Cobalt-precorrin-6B C(15)-methyltransferase | gene:cbit name:"cobalt-precorrin-6B C(15) -methyltransferase" AND reviewed:yes gene:cbit name:"cobalt-precorrin-6B C(15) -methyltransferase" AND reviewed:no |
| cbiX | Sirohydrochlorin cobaltochelatase | gene:cbix name:"sirohydrochlorin cobaltochelatase" AND reviewed:yes gene:cbix name:"sirohydrochlorin cobaltochelatase" AND reviewed:no |
| cbiZ | Adenosylcobinamide amidohydrolase | gene:cbiz name:"adenosylcobinamide amidohydrolase" AND reviewed:yes gene:cbiz name:"adenosylcobinamide amidohydrolase" AND reviewed:no |
| btuB | Vitamin B_12_ transporter BtuB | gene:btub name:"vitamin B_12_ transporter btub" AND reviewed:yes gene:btub name:"vitamin B_12_ transporter btub" NOT name:"tonb-dependent receptor" AND reviewed:no |
| btuC | Vitamin B_12_ import system permease protein BtuC | gene:btuc (name:"vitamin B_12_ import system permease protein btuc" OR name:"cobalamin import system permease protein btuc") AND reviewed:yes gene:btuc (name:"vitamin B_12_ import system permease protein btuc" OR name:"cobalamin import system permease protein btuc") AND reviewed:no |
| btuD | Vitamin B_12_ import ATP-binding protein BtuD; | gene:btud (name:"vitamin B_12_ import ATP-binding protein btud" OR name:"cobalamin import ATP-binding protein btud") AND reviewed:yes gene:btud (name:"vitamin B_12_ import ATP-binding protein btud" OR name:"cobalamin import ATP-binding protein btud") NOT name:"aaa atpase" AND reviewed:no |
| btuF | Vitamin B_12_-binding protein | gene:btuf name:"vitamin B_12_-binding protein" NOT name:"cobalamin-binding protein" AND reviewed:yes gene:btuf name:"vitamin B_12_-binding protein" NOT name:"iron abc transporter substrate-binding protein" AND reviewed:no |
| btuR | Cob(I)alamin adenosyltransferase | gene:btur NOT gene:coba name:"cob(I)alamin adenosyltransferase" AND reviewed:no |
| eutT | Ethanolamine utilization cobalamin adenosyltransferase | gene:eutt name:"ethanolamine utilization cobalamin adenosyltransferase" AND reviewed:yes gene:eutt name:"ethanolamine utilization cobalamin adenosyltransferase" AND reviewed:no |
| pduO | Corrinoid adenosyltransferase | gene:pduo name:"corrinoid adenosyltransferase" |
| pduS | Cob(II)alamin reductase | gene:pdus name:"cob(II)alamin reductase" |
| fre | NAD(P)H-flavin reductase | gene:fre name:"NAD(P)H-flavin reductase" AND reviewed:yes gene:fre name:"NAD(P)H-flavin reductase" AND reviewed:no |
| ubiB | Aquacobalamin reductase | gene:ubib name:"aquacobalamin reductase" AND reviewed:yes gene:ubib name:"aquacobalamin reductase" AND reviewed:no |
| bluB | 5,6-dimethylbenzimidazole synthase | gene:blub name:"5,6-dimethylbenzimidazole synthase" AND reviewed:yes gene:blub name:"5,6-dimethylbenzimidazole synthase" AND reviewed:no |
