# Supplementary figures and images for: VB12Path for Accurate Metagenomic Profiling of Microbially Driven Cobalamin Synthesis Pathways
Source: mSystems. 2021 Jun 1;6(3):e00497-21. doi: 10.1128/mSystems.00497-21 (PMC8269236; doi:10.1128/mSystems.00497-21)

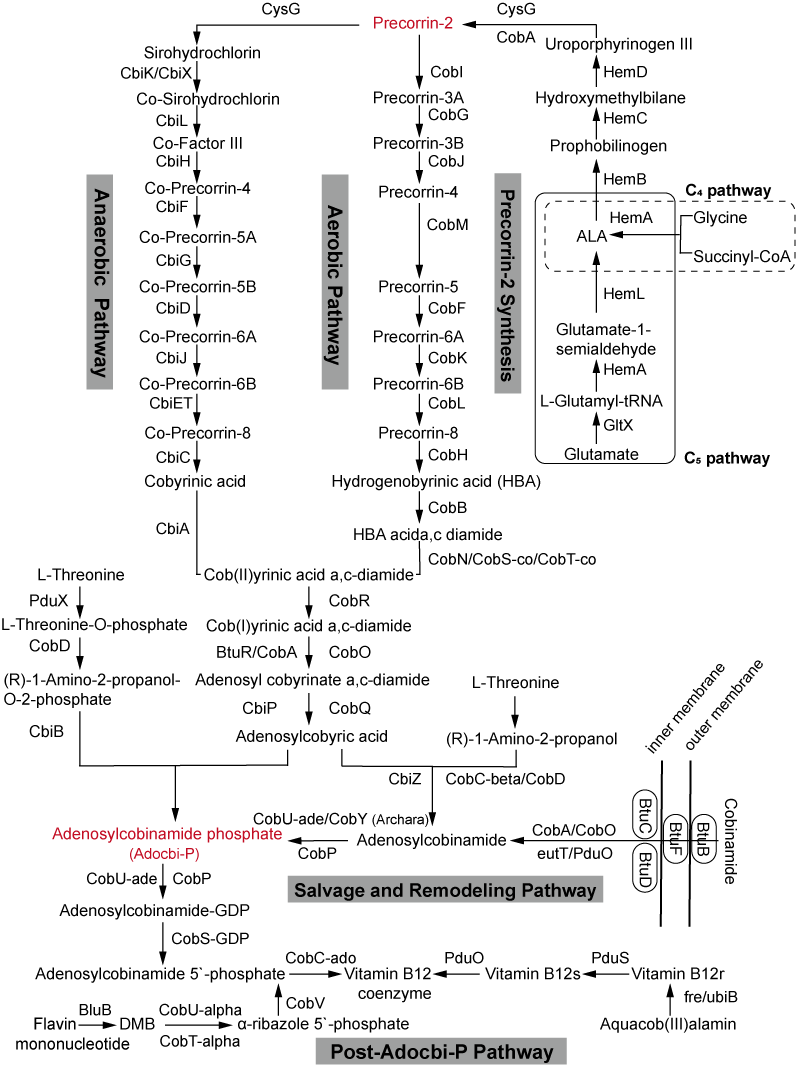

Supplement: FIG S1 [file msystems.00497-21-sf001.tif]

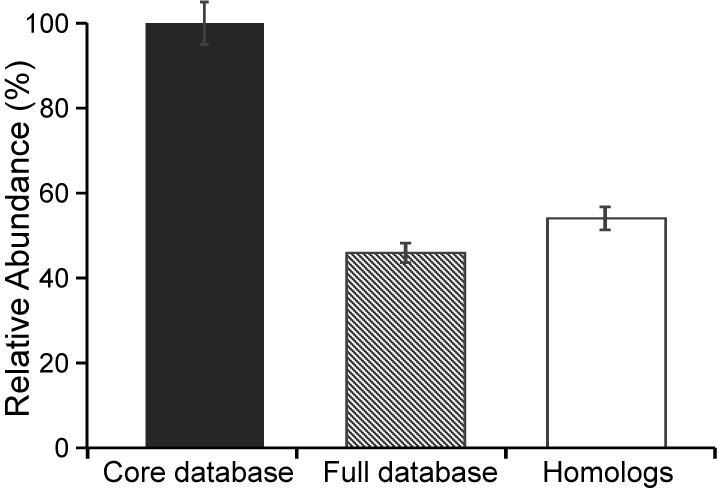

Supplement: FIG S2 [file msystems.00497-21-sf002.tif]

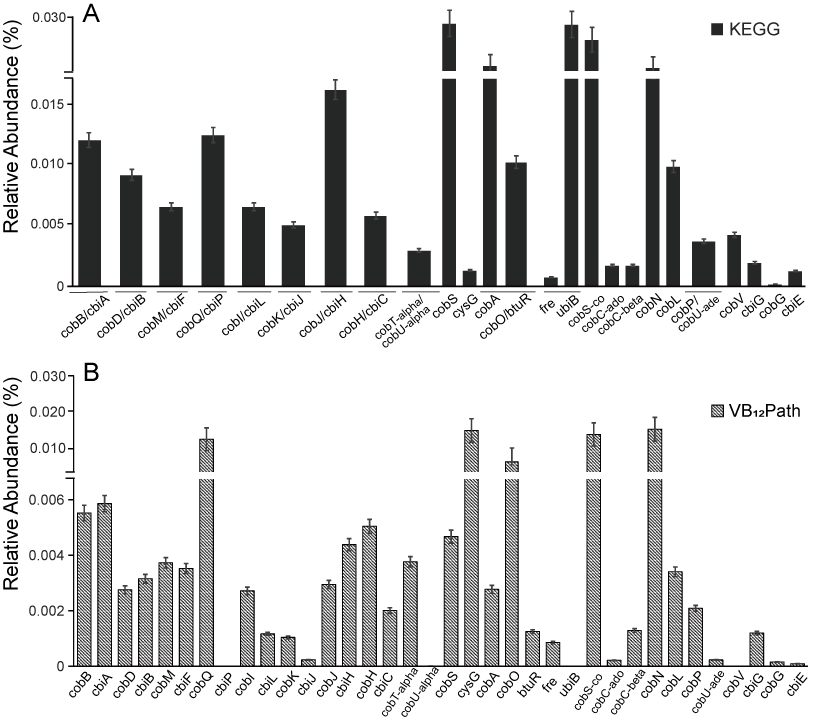

Supplement: FIG S3 [file msystems.00497-21-sf003.tif]

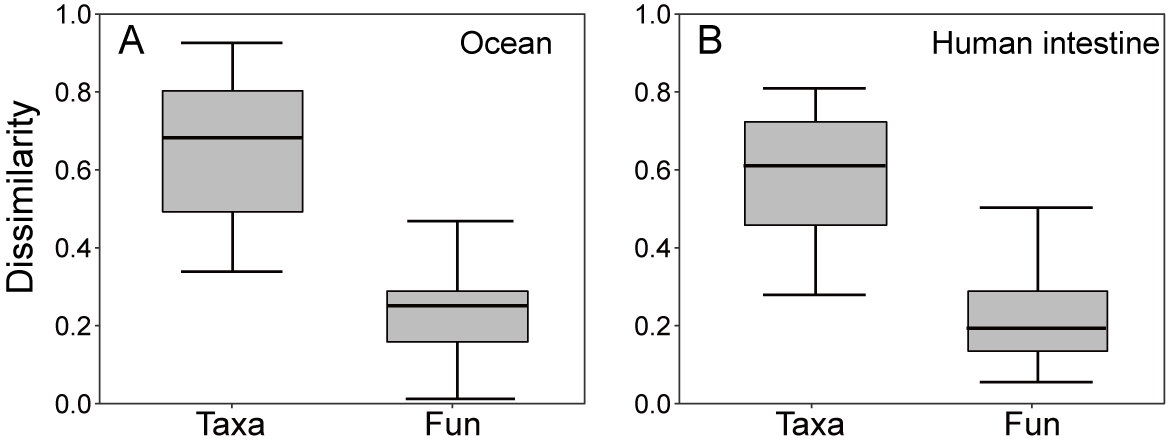

Supplement: FIG S4 [file msystems.00497-21-sf004.tif]
